# NRXN3 regulates pyroptosis in intrahepatic cholangiocarcinoma via mediating the phospho-dependent ubiquitination and degradation of caspase-3

**DOI:** 10.1016/j.jare.2025.04.040

**Published:** 2025-05-03

**Authors:** Tao Zhou, Yaodong Zhang, Tianlin Wang, Jiang Chang, Wangjie Jiang, Yananlan Chen, Shenye Shao, Ruixiang Chen, Jifei Wang, Yirui Wang, Changxian Li, Xiangcheng Li

**Affiliations:** a Hepatobiliary Center, The First Affiliated Hospital of Nanjing Medical University, Key Laboratory of Liver Transplantation, Chinese Academy of Medical Sciences, NHC Key Laboratory of Living Donor Liver Transplantation, Nanjing Medical University, Nanjing, Jiangsu Province, China; b Gusu School of Nanjing Medical University, The Affiliated Suzhou Hospital of Nanjing Medical University, Suzhou, Jiangsu Province, China; c Wuxi Medical Center of Nanjing Medical University, The Affiliated Wuxi People’s Hospital of Nanjing Medical University, Wuxi, Jiangsu Province, China; d Jiangsu Key Laboratory of Cancer Biomarkers, Prevention and Treatment, Collaborative Innovation Center for Cancer Personalized Medicine, Nanjing Medical University, Nanjing, Jiangsu Province, China

**Keywords:** Pyroptosis, Gemcitabine, Caspase-3, Phosphorylation, Ubiquitination, Intrahepatic cholangiocarcinoma

## Abstract

**Introduction:**

Intrahepatic cholangiocarcinoma (ICC) remains one of the most lethal malignancies with an increasing incidence worldwide. Gemcitabine has been considered the standard first-line chemotherapeutic agent for ICC but the therapeutic response is unsatisfactory due to the development of chemoresistance. Caspase-3-mediated pyroptosis has been reported to play significant roles in chemotherapeutic response but the relevant therapeutic strategy remains unstated due to the unclear molecular mechanisms under pyroptosis in ICC.

**Objectives:**

This study was designed to comprehensively explore the crucial role and underlying mechanisms of NRXN3 in pyroptosis and chemosensitivity of ICC.

**Methods:**

We performed genome-scale CRISPR-Cas9 screen integrated with transcriptomic analysis to identify key regulators of pyroptosis and gemcitabine sensitivity in ICC. In vitro and in vivo experiments were employed to investigate the effects of NRXN3 on gemcitabine-induced pyroptosis. RNA-seq and IP-MS were conducted to explore the mechanisms of NRXN3-regulated pyroptosis and chemosensitivity.

**Results:**

NRXN3 was identified to be a critical contributor to pyroptosis and chemosensitivity in ICC. Low NRXN3 expression correlates with poor prognosis and worse therapeutic response. Mechanistically, NRXN3 competitively blocks caspase-3 binding to the RSK1 serine/threonine-protein kinase, thereby inhibiting RSK1-dependent phosphorylation of caspase-3 at T152. Inhibition of caspase-3 phosphorylation impairs its interaction with the ubiquitin ligase component FBXO1 and enhances its stability, thus facilitating caspase-3/GSDME-dependent pyroptotic cell death and chemosensitivity. Furthermore, administration of an RSK1 inhibitor or caspase-3 activator boosts the efficacy of gemcitabine in murine models of ICC.

**Conclusion:**

NRXN3 plays a crucial role in maintaining chemotherapy-induced pyroptosis in ICC. Targeting the NRXN3/RSK1/FBXO1/caspase-3 axis emerges as a promising strategy for ICC treatment, with the potential to improve chemosensitivity and survival.

## Highlights

• NRXN3 is identified as a critical contributor to pyroptosis and chemosensitivity in intrahepatic cholangiocarcinoma (ICC).
• NRXN3 competitively blocks caspase-3 binding to the RSK1 serine/threonine-protein kinase.
• RSK1-dependent phosphorylation of caspase-3 at T152 promotes the ubiquitination and degradation of caspase-3.
• Administration of BI-D1870 or Raptinal boosts the efficacy of gemcitabine in murine models of ICC.

## Graphical abstract

**Figure:**
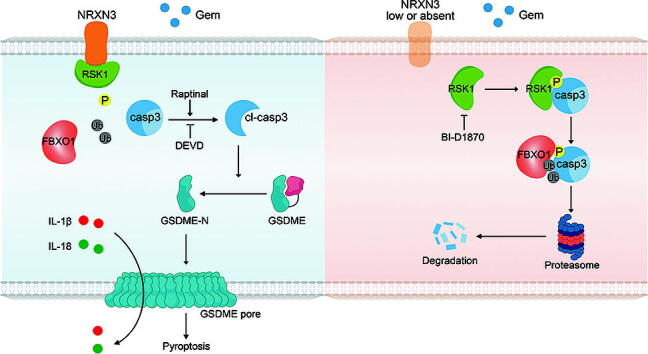

## Introduction

Intrahepatic cholangiocarcinoma (ICC), the second most common primary liver tumor, remains a lethal malignancy for the majority of patients, with a gradually increasing incidence worldwide. Approximately 70 %-80 % patients present with unresectable disease at diagnosis, for whom systemic therapy is the only treatment option available [1]. Gemcitabine has been considered the standard first-line chemotherapeutic agent but the therapeutic effect is unsatisfactory due to chemoresistance. Despite combination with cisplatin, the median overall survival remains dismally limited to approximately one year [2]. Therefore, the quest for strategies to enhance gemcitabine sensitivity is crucial for extending the survival of patients.

Pyroptosis, a gasdermin (GSDM)-dependent programmed cell death, is characterized by chromatin condensation, cell swelling and ballooning, membrane pore formation, blebbing, rupture and release of cellular contents. Self-inhibited GSDMs is cleaved by active caspases to release a perforative N-terminus which initiates the pyroptotic process [3,4]. Caspase-3/GSDME pyroptotic pathway has been reported to play critical roles in chemotherapy-induced cell death. Wang et al. first found that chemotherapy can induce caspase-3/GSDME-mediated pyroptosis in tumor cells [5]. Recently, more studies showed that chemotherapeutic drugs can trigger pyroptosis via the caspase-3/GSDME pathway in tumor cells, further underscoring the emerging trend of inducing pyroptosis in cancer treatment strategies [[6], [7], [8], [9]]. Moreover, pyroptotic tumor cells reverse immunosuppression of the tumor microenvironment (TME) and lead to robust antitumor immunity due to the release of cellular antigens, damage-associated molecular patterns (DAMPs) and cytokines, which makes it a promising road to next-generation cancer immunotherapy [10,11].

The gene neurexin-3 (NRXN3) encodes a type-1 transmembrane protein belonging to the neurexin protein family which performs distinct functions via different molecular mechanisms. Previous studies, mostly on nervous system, discovered NRXN3 is involved in synaptic transmission, cell recognition and cell adhesion [12]. While NRXN3 is primarily studied in the context of neurological functions, evidence suggests that it may play a role in tumor progression. It is reported that NRXN3, as a tumor-suppressor, can inhibit proliferation, migration and invasion in glioma and nasopharyngeal carcinoma [[13], [14], [15]]. Also in our previous study, NRXN3 mutations were found in ICC through whole-exome sequencing, implying that alterations of NRXN3 may be involved in ICC progression [16]. Nevertheless, the function of NRXN3 in most malignancies including ICC and the underneath mechanism remain to be explored.

The development of next-generation sequencing and the utilization of multiomics help elucidate the molecular mechanisms of pyroptosis and pave the way to circumvent chemoresistance. In this study, we integrated genome-scale CRISPR-Cas9 screen with transcriptomic analysis and identified NRXN3 as a key contributor to pyroptosis and gemcitabine sensitivity in ICC. We examined the role of NRXN3 in gemcitabine-induced pyroptosis and treatment response. Through immunoprecipitation coupled with mass spectrometry (IP-MS), we identified that NRXN3 competitively binds to the RSK1 serine/threonine-protein kinase, inhibiting the phosphorylation and phospho-dependent ubiquitination of caspase-3, thereby facilitating the GSDME-dependent pyroptosis in ICC. These results highlight the post-translational modifications of caspase-3 in the regulation of pyroptosis, indicating the potential of enhancing chemosensitivity via targeting the NRXN3/RSK1/FBXO1/caspase-3 axis. Our study calls for renewed emphasis on the role of pyroptosis in the control of cancer progression and chemotherapy response.

## Materials and methods

### Animal experiments

All animal studies were conducted according to the protocol approved by the Institutional Animal Care and Use Committee of Nanjing Medical University. Six-week-old male BALB/c nude mice (GemPharmatech, China) were purchased to perform in vivo experiments. For evaluating the effects of NRXN3/RSK1/caspase-3/FBXO1 on chemotherapy response in vivo, suspensions containing 5 × 10^6^ HuCCT1 or HuCCT1-R cells that were genetically modified were injected subcutaneously into the nude mice. When the tumors became palpable, the mice were treated with placebo or gemcitabine (50 mg/kg, every 4 days, intraperitoneally), BI-D1870 (20 mg/kg, daily for 7 days, intraperitoneally), Raptinal (20 mg/kg, daily for 7 days, intraperitoneally) or their combinations. Once the placebo-treated tumors approached 1000 mm^3^, all mice were sacrificed and the tumor weight was measured.

### Whole-genome CRISPR-Cas9 knockout library screen

Human genome-scale CRISPR knockout library (Addgene#73179) was packed into lentiviral particle and transduced into HuCCT1 cells at a MOI of 0.3. The transduced cells were selected with 2 μ g/mL puromycin for 7 days. Subsequently, the cells were treated with either DMSO (Control) or Raptinal (200 nM) for 21 days. Three biological replicates of at least 2 × 10^7^ cells from each group were harvested for genomic DNA extraction. The sgRNA region was amplified by PCR and subjected to next-generation sequencing. The sequencing was analyzed using the MAGeCK algorithm.

### Human tissue samples and microarray

The tissue microarray was constructed by Outdo Biotech Company (Shanghai, China) from 120 ICC patients who underwent surgical procedures in 2006 to 2017 at The First Affiliated Hospital of Nanjing Medicine University. The expression of NRXN3 was evaluated using a grade semiquantitative scoring system. The intensity was classified as negative (0), weak (1), moderate (2), or strong (3), and the density of positive cells in the target region was scored as follows: <5% (0), 5–25 % (1), 26–50 % (2), 51–75 % (3), and > 75 % (4). The overall score was calculated by multiplying intensity score and density score. Each microarray tissue point was scored by two independent pathologists, and the average score was taken as the final score. Tumors with scores of ≤ 4 were defined as low expression, while those with higher scores were categorized as high expression. The patients were followed up regularly until death or October 25, 2019. The Ethics Committee of The First Affiliated Hospital of Nanjing Medical University approved the use of clinical samples, and written informed patient consent was obtained in accordance with regional regulations.

### Antibodies and reagents

The antibodies and reagents used in this study are listed in Supplementary Table. S2.

### Cell culture

Human ICC cell lines HuCCT1 and HCCC9810, as well as Human HEK-293 T, were cultured in DMEM medium supplemented with 10 % fetal bovine serum and 1 % penicillin/streptomycin. Gemcitabine-resistant cell lines HuCCT1-R and HCCC9810-R were established by exposing to increasing doses of gemcitabine for 9 months and then persistently cultured in gemcitabine-containing medium.

### Immunoprecipitation and mass spectrometry

Cells were harvested using NP-40 lysis buffer and immunoprecipitated with the indicated antibodies or normal IgG. Successful immunoprecipitation was verified using silver staining and western blot. The immunoprecipitant product was then subjected to western blot for Co-IP analysis or liquid chromatography with tandem mass spectrometry for proteomics analysis (BGI Genomics, Shenzhen, China).

### RNA sequencing

Total RNA was extracted from the indicated cells and the concentration and purity were checked by NanoDrop 2000 Nucleic Acid and Protein Analyzer (Thermofisher, MA, USA). The procedure of RNA sequencing was previously described [17]. Data analysis was performed by Beijing Biomarker Technologies (Shanghai, China).

### RNA Interference, Plasmids, CRISPR/Cas9 Editing, and lentivirus infection

The plasmids and siRNAs were constructed by Corues Biotechnology (Nanjing, China) and transfected using Lipofectamine 3000 Transfection Kit (Invitrogen, USA) according to the manufacturer’s instruction. CRISPR/Cas9 technology was employed to knockout NRXN3 in ICC cells and the organoid. RT-qPCR, western blot or sanger sequencing were used to verify transfection efficiency. The lentiviruses were constructed by OBiO Technology (Shanghai, China). Cells were seeded in 6-well plates and then infected with lentivirus according to the manufacturer’s protocol. After 48 h, the medium was replaced with complete medium. The infected cells were treated with 10 mg/ml puromycin in order to select stable transfected cells. The related sequences are listed in Supplementary Table. S3.

### Statistical analysis

The results are represented as mean ± SD or mean ± SEM of at least three independent experiments. All statistics analyses were performed in GraphPad Prism 8.0 and SPSS 24.0 software. Significance was calculated using two-tailed unpaired Student’s *t*-test for comparations between two groups with P < 0.05 representing statistical significance. Survival curves were generated using Kaplan-Meier method and significance was calculated with Log-rank test.

More detailed methods are provided in Supplementary Methods.

## Results

### Integrated whole-genome CRISPR screen with transcriptomic analysis to reveal critical contributors to both pyroptosis and chemosensitivity in ICC

**Fig. 1:**
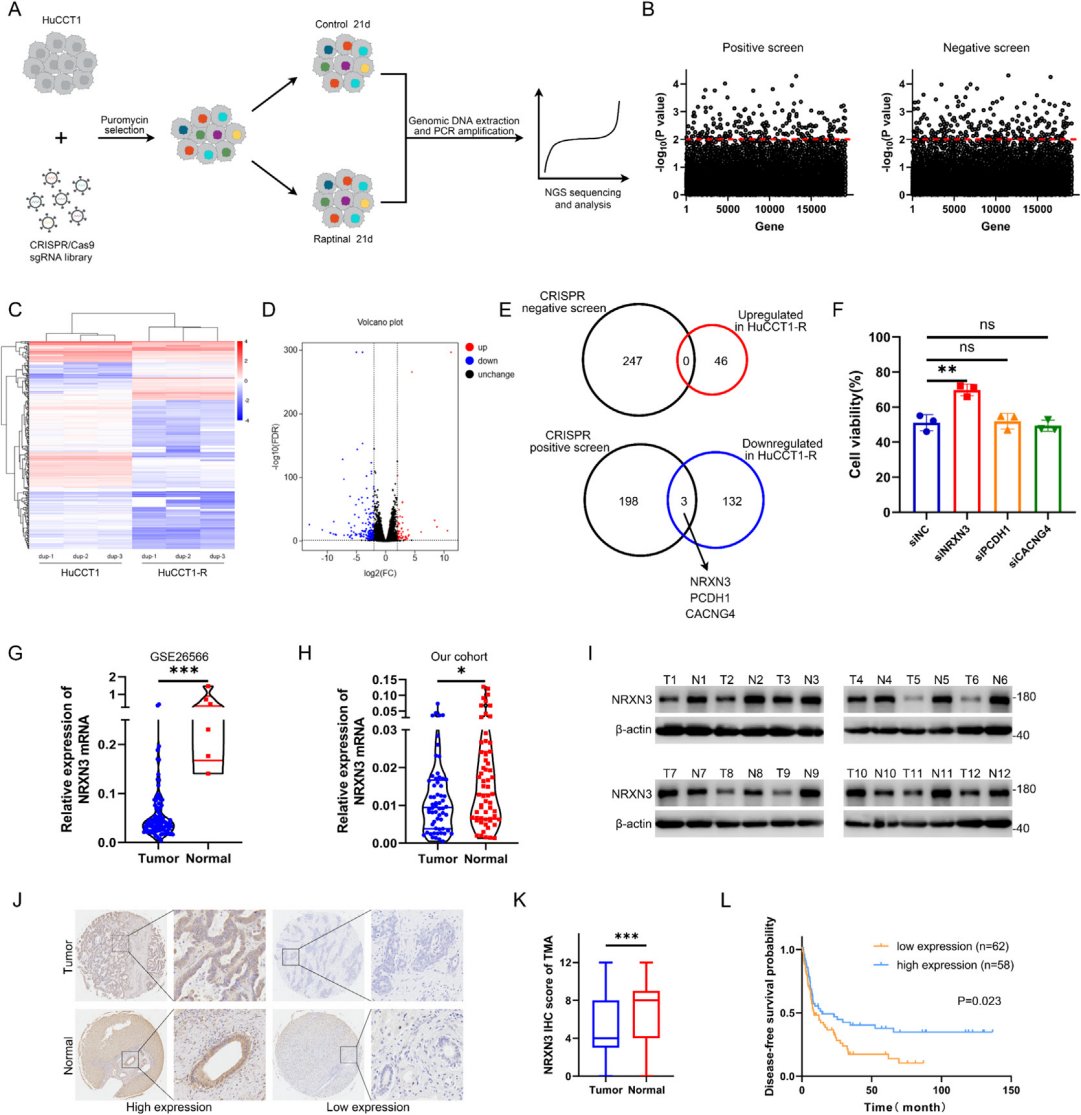
**Integrated whole-genome CRISPR screen with transcriptomic analysis to reveal critical contributors to both pyroptosis and chemosensitivity in ICC.** (A) Schematic representation of the whole-genome CRISPR screen workflow in the ICC cell line, HuCCT1. (B) Scatter plots depicting results for Raptinal positively and negatively selected hits in the CRISPR screen. The hits above the red dashed line (p < 0.01) are statistically significant. (C) Heatmap of differentially expressed genes in the transcriptomic analysis of HuCCT1 and HuCCT1-R cells. (D) Volcano plot of differentially expressed genes from the transcriptomic analysis of HuCCT1 and HuCCT1-R cells. (E) Venn diagram depicting the intersection between whole-genome CRISPR screen and transcriptomic analysis. (F) Cell viability of HuCCT1 cells transfected with empty vector or siRNA targeting NRXN3, PCDH1 or CACNG4 after treatment with gemcitabine for 48 h. (G) Violin plot shows the relative expression of NRXN3 mRNA in cholangiocarcinoma (n = 104) versus normal intrahepatic bile duct (n = 6) samples from GSE26566. (H) Violin plot shows the relative expression of NRXN3 mRNA in ICC versus normal intrahepatic bile duct samples of our cohort (n = 60). (I) Immunoblotting analysis of relative expression of NRXN3 protein in ICC versus normal intrahepatic bile duct samples of our cohort. (J) Representative immunohistochemical staining images of NRXN3 in ICC and normal tissues from our tissue microarrays. (K) Box plot shows immunohistochemical scores of NRXN3 in ICC and normal tissues from our tissue microarrays. (L) Correlation between NRXN3 expression and disease-free survival in patient with ICC. *P < 0.05, **P < 0.01, and ***P < 0.001. (F-H, K) Student’s *t* test. (L) Log-rank (Mantel-Cox) test.

Pyroptosis is a recently identified programmed cell death, which was initialized by activation of various caspases, leading to cleavage and multimerization of various GSDM family members which cause cell perforation and death. Raptinal is an agent that can activate caspase-3 and cleave GSDME to induce pyroptosis [18]. We observed pyroptotic membrane ballooning in HuCCT1 cells after treatment with Raptinal, confirming that Raptinal can induce pyroptosis in ICC cells (Fig. S1A). Besides, we performed Raptinal median inhibitory concentration (IC_50_) experiments of HuCCT1 cell line (Fig. S1B). To elucidate the mechanism and explore the clinical potential of pyroptosis in ICC, we performed genome-scale CRISPR knockout library screen with Raptinal in HuCCT1 cell line (Fig. 1A). Human CRISPR knockout library that containing 76,441 gRNAs targeting 19,114 genes was used to generated a mutant cell pool. The mutant cells were treated with either Raptinal or DMSO (control) for 21 days and then subjected to next-generation sequencing (NGS) to analyze the differential sgRNA between two groups. The total sequencing reads and mapping ratio of the screen were shown in Fig. S1C. In the presence of Raptinal, cells carrying sgRNAs targeting genes conferring resistance to pyroptosis will be eliminated and the corresponding sgRNAs will be depleted, which could be determined by sequencing. Therefore, negative screen was used to identify potential genes conferring resistance to pyroptosis. On the contrary, positive screen was applied to identify potential genes that facilitate pyroptosis (Fig. 1B).

Pyroptosis has been reported to play significant roles in chemotherapeutic response and considered as a promising therapeutic strategy for the malignancies exhibiting chemoresistance. To identify which genes in the pyroptosis-related CRISPR screen that might influence chemosensitivity, we integrated the CRISPR screen data with transcriptomic data of gemcitabine-sensitive and gemcitabine-resistant ICC cell lines. The gemcitabine-resistant ICC cell lines were constructed to investigate chemoresistance of ICC (Fig. S1D). The sensitive HuCCT1 and resistant HuCCT1-R were subjected to transcriptomic sequencing as well as KEGG pathway analysis and GSEA analysis (Fig. 1C, Fig. S1E-F). These genes upregulated in HuCCT1-R were regarded as potential contributors to gemcitabine resistance while these downregulated were regarded as potential contributors to gemcitabine sensitivity (Fig. 1D). By integrating CRISPR negative screen and HuCCT1-R upregulated genes, we did not identify any gene that potentially confer resistance to both pyroptosis and gemcitabine treatment. However, by the integrated analysis of CRISPR positive screen and HuCCT1-R downregulated genes, three genes were identified to potentially promote both pyroptosis and gemcitabine sensitivity in ICC (Fig. 1E). Then the three genes were assumed to facilitate chemosensitivity through enhancing pyroptosis. To validate the effects of NRXN3, PCDH1 and CACNG4 on gemcitabine sensitivity in ICC, we knockdown them respectively using siRNA (Fig. S1G). We observed that knockdown of NRXN3 significantly enhanced ICC resistance to gemcitabine (Fig. 1F). Therefore, our further study focused on the clinical relevance and molecular mechanism of NRXN3.

To elucidate the clinical relevance of NRXN3, we explored the expression profile of NRXN3 in GEO database and our patient cohorts. The analysis of a GEO dataset (GSE26566) which contains 104 cholangiocarcinoma samples and 6 normal intrahepatic bile duct samples showed that NRXN3 mRNA expression was significantly downregulated in tumors compared with normal tissues (Fig. 1G). RT-qPCR analysis and immunoblotting analysis of our sample cohorts confirmed the downregulation of NRXN3 mRNA and protein in ICC (Fig. 1H-I). Furthermore, we performed immunohistochemical staining (IHC) of the tissue microarrays containing 120 ICC tissues and 99 corresponding adjacent normal tissues (Fig. 1J). The IHC scores are significantly higher in the normal group than the tumor group (Fig. 1K). The 120 ICC patients were stratified as either low or high expression according to the IHC scores and the association between NRXN3 expression and their clinicopathological characteristics was analyzed. Low NRXN3 expression was significantly positively correlated with higher serum CA19-9 level (*p* = 0.013) and higher rate of recurrence (*p* = 0.028) (Supplementary Table. S1). Further univariable analyses showed that NRXN3 expression, clinical stage, lymph node status and histological grade are significantly associated with the disease-free survival of ICC patients. Among these factors, NRXN3 expression and histological grade stand out as independent prognostic factors in multivariable analysis (Table. 1). The Kaplan-Meier survival curves shows that ICC patients with high NRXN3 expression exhibited longer postoperative disease-free survival than those with low expression (Fig. 1L). In ICC patients treated with adjuvant chemotherapy, high NRXN3 expression significantly correlated with better disease-free survival (Fig. S1H). Considering the extensive roles of pyroptosis in various antitumor therapies, we performed a survival analysis using Kaplan-Meier Plotter database to uncover the significance of NRXN3. The results showed that high NRXN3 expression was associated with significantly improved overall survival in chemotherapy-treated breast cancer and progression-free survival in sorafenib-treated liver cancer (Fig. S1I-J). And in immunotherapy-treated melanoma, esophageal adenocarcinoma and glioblastoma, patients with high NRXN3 expression exhibited significantly prolonged overall survival or progression-free survival (Fig. S1K-M). Further studies are needed to elucidate the role of NRXN3 in these malignancies. Together, these results suggest that NRXN3 may function as a key contributor to both pyroptosis and gemcitabine sensitivity in ICC.

**Table 1:** Univariate and multivariate analyses of the prognostic factors in ICC patients.

**Variable**	**Univariate analysis**			**Multivariate analysis**		
	**HR**	**95 % CI**	**P value**	**HR**	**95 % CI**	**P value**
Gender,male/female	0.872	0.561–1.355	0.543			
Age,≤60/＞60	0.929	0.591–1.462	0.751			
CA199(U/L),≤37/＞37	1.884	0.842–4.211	0.123			
CEA(ng/ml),≤5/＞5	1.370	0.642–2.920	0.416			
AFP(ng/ml),≤20/＞20	1.860	0.639–5.407	0.255			
Diameter(cm)，≤3/＞3	1.172	0.662–2.074	0.587			
Histological grade,I,I-II,II/II-III,III	1.975	1.211–3.221	0.006*	2.106	1.276–3.477	0.004*
Tumor thrombus,absent/present	1.632	0.940–2.834	0.082			
Nerve invasion,absent/present	1.122	0.663–1.897	0.668			
Lymph nodes,negative/positive	1.952	1.121–3.397	0.018*	2.140	0.606–7.557	0.237
Clinical stage,I-II/III-IV	1.700	1.017–2.844	0.043*	0.853	0.261–2.787	0.793
Surgical margin，R0/R1,R2	1.469	0.707–3.055	0.303			
NRXN3 expression,low/high	0.596	0.380–0.934	0.024*	0.611	0.377–0.992	0.046*

### NRXN3 facilitates gemcitabine-induced pyroptosis and chemosensitivity in ICC

**Fig. 2:**
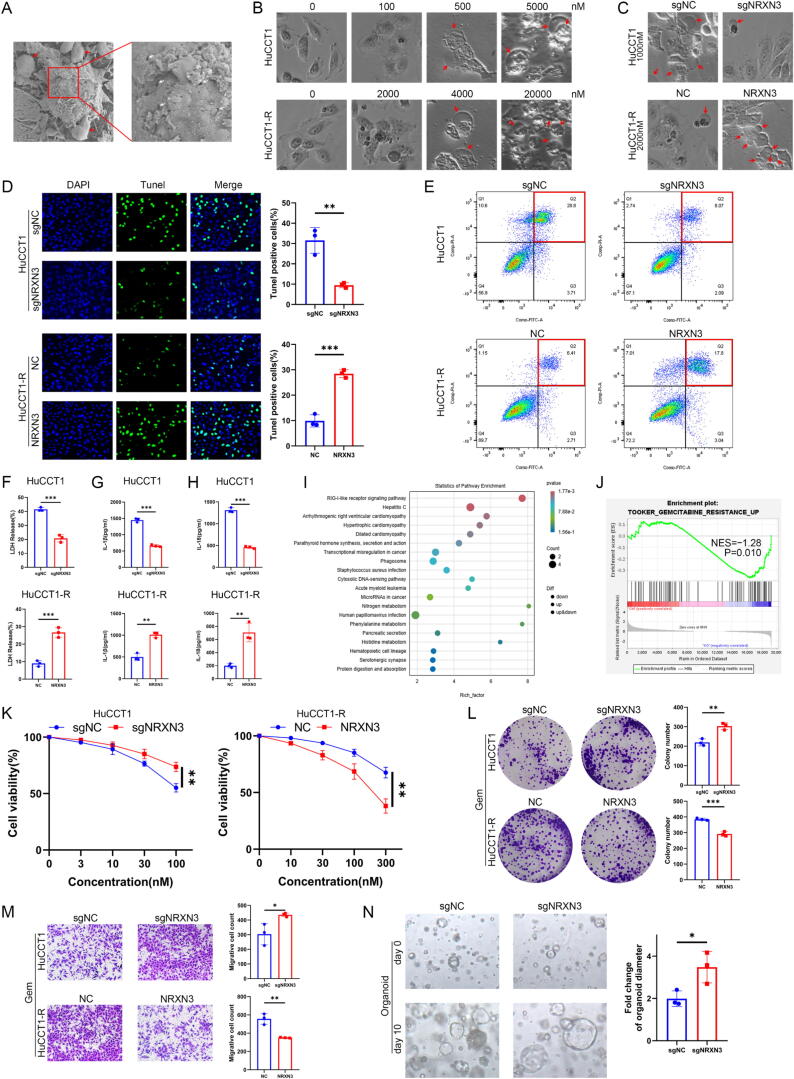
**NRXN3 facilitates gemcitabine-induced pyroptosis and chemosensitivity in ICC.** (A) Representative scanning electron microscopy images of HuCCT1 cells treated with gemcitabine. Red arrows indicate bubble-like protrusions on the membrane and white arrows indicate pits and pores of varying sizes. (B) Representative high-throughput bright-field images of HuCCT1 and HuCCT1-R treated with escalated doses of gemcitabine. Red arrows indicate characteristic ballooning in cell membranes. (C) Representative bright-field images of HuCCT1 cells transduced with vector or sgNRXN3 lentiviruses, and HuCCT1-R cells transduced with vector or NRXN3-overexpressing lentiviruses after treatment of gemcitabine. Red arrows indicate characteristic ballooning in cell membranes. (D) Tunel assays of HuCCT1 and HuCCT1-R cells after treatment with gemcitabine. (E) Flow cytometry analysis of HuCCT1 and HuCCT1-R cells treated with gemcitabine and stained with Annexin V-FITC/PI. Pyroptotic cell death: Annexin V-FITC+/PI+; non-pyroptotic cell death: Annexin V-FITC+/PI- and Annexin V-FITC-/PI+; live cell: Annexin V-FITC-/PI-. (F) LDH release assays of HuCCT1 and HuCCT1-R cells after treatment with gemcitabine (5000 nM). (G) Measurement of IL-1β release in HuCCT1 and HuCCT1-R cells after treatment with gemcitabine. (H) Measurement of IL-18 release in HuCCT1 and HuCCT1-R cells after treatment with gemcitabine. (I) KEGG pathway enrichment analysis of the transcriptomic data from HuCCT1 cells transduced with vector or sgNRXN3 lentiviruses. (J) Genes associated with upward gemcitabine resistance are enriched in HuCCT1 cells transduced with sgNRXN3 lentiviruses (KO). (K) Cell viability of HuCCT1 and HuCCT1-R cells treated with gemcitabine at different dose for 48 h. (L) Colony formation assays of HuCCT1 and HuCCT1-R cells in low concentration of gemcitabine. (M) Migration assays of HuCCT1 and HuCCT1-R cells in the presence of gemcitabine. (N) Representative images and diameter fold change of the patient-derived ICC organoid at day 0 and day 10 after treatment with gemcitabine. *P < 0.05, **P < 0.01, and ***P < 0.001. (D, F-H, K-N) Student’s *t* test.

First of all, we validated that gemcitabine treatment could trigger pyroptosis in ICC cells. Scanning electron microscopy of gemcitabine-treated ICC cells showed bubble-like protrusions on the membrane and pits and pores of varying sizes (Fig. 2A). Through the phase-contrast microscope, we observed the morphological changes of ICC cells in different gemcitabine concentration. With the increase of gemcitabine concentration, cells swelled in accompany with characteristic pyroptotic membrane ballooning and rupture (Fig. 2B). To determine the role of NRXN3 in gemcitabine-induced pyroptosis and chemosensitivity of ICC, we genetically modified NRXN3 expression in ICC cell lines and a patient-derived ICC organoid. The endogenous expression of NRXN3 in the cell lines was detected using RT-qPCR and western blotting, which confirmed that NRXN3 was downregulated in gemcitabine resistant ICC cells (Fig. S2A-B). We constructed stable NRXN3-knockout HuCCT1 and HCCC9810 cell line and organoid with CRISPR/Cas9 editing and stable NRXN3-overexpression HuCCT1-R and HCCC9810-R cell line with lentivirus infection. The efficiency of gene editing was verified by RT-qPCR, western blotting or sanger sequencing (Fig. S2C-E).

Then we performed a range of in vitro assays with the genetically modified cells to explore the functional role of NRXN3 in gemcitabine-induced pyroptosis. Phase-contrast microscopy showed that knockout of NRXN3 inhibited gemcitabine-induced pyroptosis while NRXN3 overexpression facilitated gemcitabine-induced pyroptosis (Fig. 2C). In Tunel assays, the percentage of Tunel-positive cells induced by gemcitabine decreased in NRXN3-knockout group but increased in NRXN3-overexpression group (Fig. 2D and S3A). Further flowcytometry assays confirmed that NRXN3 knockout or overexpression greatly affected pyroptotic cell death (Annexin V-FITC^+^/PI^+^). Knockout of NRXN3 alleviated pyroptotic cell death while NRXN3 overexpression aggravated pyroptotic cell death (Fig. 2E and S3B). LDH release assays and detection of IL-1β and IL-18 also verified that gemcitabine-induced pyroptosis in ICC was ameliorated by NRXN3 knockout but exacerbated by NRXN3 overexpression (Fig. 2F-H and S3C-E). Next, we performed transcriptomic sequencing with gemcitabine-treated NRNX3-knockout and control HuCCT1 cells. The KEGG pathway analysis revealed an enrichment in inflammation-related signaling pathways and processes such as RIG-I-like receptor signaling pathway, phagosome and cytosolic DNA-sensing pathway, which again verified the critical role of NRXN3 in pyroptosis (Fig. 2I). Furthermore, GSEA revealed that in NRNX3-knockout HuCCT1 cells the genes associated with upward gemcitabine resistance were significantly enriched (Fig. 2J). These data implied that NRXN3 might affect ICC resistance to gemcitabine through regulation of pyroptosis.

Subsequently, we detected the influence of NRXN3 on gemcitabine sensitivity in ICC. We observed that knockout of NRXN3 augmented resistance to gemcitabine in HuCCT1 and HCCC9810 cells, while NRXN3 overexpression reduced resistance to gemcitabine in the corresponding resistant HuCCT1-R and HCCC9810-R cells (Fig. 2K and S3F). In the presence of gemcitabine, NRXN3 knockout increased the colony formation number of the sensitive ICC cells but NRXN3 overexpression decreased the colony formation number of the resistant ICC cells (Fig. 2L and S3G). Besides, the migration of the sensitive cells was enhanced by NRXN3 knockout, whereas the migration of the resistant cells was suppressed by NRXN3 overexpression (Fig. 2M and S3H). Moreover, knockout of NRXN3 significantly increased the ICC organoid size after 10 days of treatment with gemcitabine (Fig. 2N). Collectively, the data above suggested that NRXN3 was able to facilitate gemcitabine-induced pyroptosis and chemosensitivity in ICC.

### NRXN3 regulates gemcitabine-induced pyroptosis in ICC cells via blocking caspase-3 interaction with RSK1 and maintaining the stability of caspase-3

**Fig. 3:**
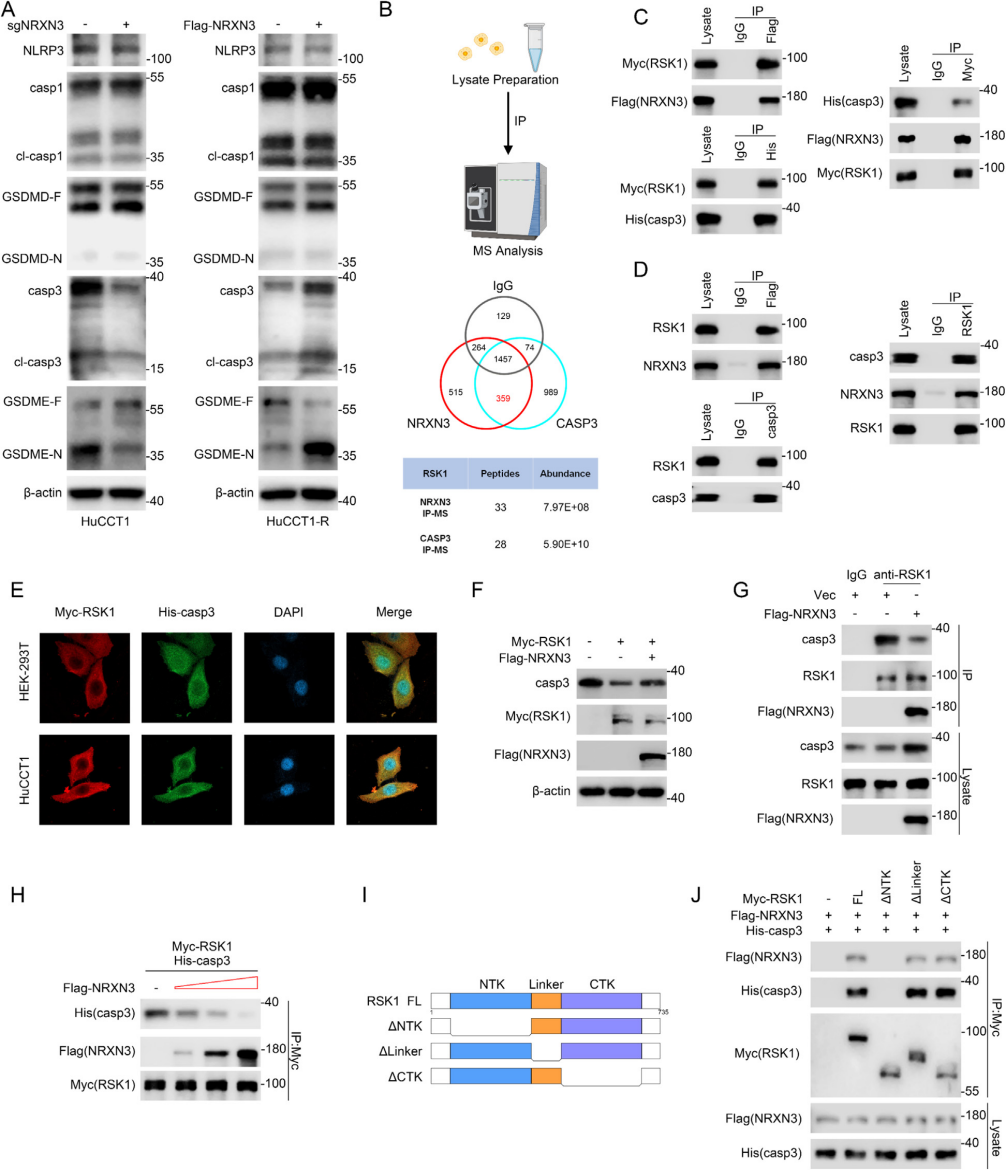
**NRXN3 regulates gemcitabine-induced pyroptosis in ICC cells via blocking caspase-3 interaction with RSK1 and maintaining the stability of caspase-3.** (A) Immunoblotting analysis of the pyroptosis-related proteins in ICC cells with NRXN3 knockout or overexpression. (B) IP-MS analysis of Flag-NRXN3-overexpressing HuCCT1 cells using anti-Flag, anti-caspase-3 (CASP3) antibodies and non-specific normal IgG. Unique peptide numbers and abundance of RSK1 are shown. (C) HEK-293 T cells were co-transfected with Flag-NRXN3, Myc-RSK1 and His-caspase-3 for 48 h. Cell lysates were analyzed by IP and IB as indicated. (D) HEK-293 T cell lysates were subjected to IP and IB as indicated. Flag-NRXN3-expressing HEK-293 T cell lysates were subjected to IP using anti-Flag antibody. (E) Immunofluorescence analysis of Myc-RSK1 and His-caspase-3 in Myc-RSK1- and His-caspase-3-expressing HEK-293 T and HuCCT1 cells. (F) HEK-293 T cells were co-transfected with Myc-RSK1 and Flag-NRXN3 or vector as indicated for 48 h, followed by IB analysis. (G) HuCCT1-R cells transduced with vector or Flag-NRXN3-expressing lentiviruses were subjected to anti-RSK1 immunoprecipitation and IB. (H) HEK-293 T cells were singly transfected with Flag-NRXN3, Myc-RSK1 and His-caspase-3. Myc-RSK1- and His-caspase-3-expressing lysates were added to all conditions in equal amount. Flag-NRXN3-expressing lysate was excluded from the first condition and added in incremental amounts to the last three conditions. Lysates were subjected to anti-Myc immunoprecipitation and IB. (I) Schematic representation of full length or various truncated RSK1. (J) HEK-293 T cells were co-transfected with Flag-NRXN3, His-caspase-3 and vector or the indicated Myc-tagged RSK1 plasmids. Lysates were subjected to anti-Myc immunoprecipitation and IB.

We detected the typical pyroptotic GSDMD and GSDME pathways in gemcitabine-treated ICC cells with NRXN3 knockout or overexpression. The results showed no significant changes in the classical NLRP3/caspase-1/GSDMD pathway. However, the protein level of caspase-3 was significantly downregulated by NRXN3 knockout, and consequently GSDME cleavage was attenuated. In NRXN3-overexpression cells, caspase-3 protein level was upregulated and GSDME was significantly cleaved (Fig. 3A). However, RT-qPCR analysis showed that the mRNA of caspase-3/GSDME did not change as the protein did (Fig. S4A). These results indicated that caspase-3 expression was regulated by NRXN3 at the protein level while the further co-immunoprecipitation (Co-IP) assays suggested that NRXN3 failed to interact with caspase-3 (Fig. S4B). To explore the mechanism of NRXN3 regulating caspase-3, we performed immunoprecipitation (IP) in Flag-NRXN3-overexpressing HuCCT1 cells using anti-Flag and anti-caspase-3 antibodies, with non-specific normal IgG as negative control. The IP products were subjected to mass spectrometry (MS) analysis to identify the potential bridge molecules between NRXN3 and caspase-3 (Fig. 3B). RSK1, a serine/threonine-protein kinase that is activated by ERK or PDK1 and acts as a downstream effector of MAPK, was identified as a potential interacting partner between NRXN3 and caspase-3. Co-IP assays verified that exogenous and endogenous RSK1 interacted with NRXN3 and caspase-3 respectively (Fig. 3C-D). The immunofluorescence staining demonstrated that RSK1 co-localized with caspase-3 in the cytoplasm (Fig. 3E). In Co-IP assays, the N-terminus of NRXN3 failed to co-precipitate RSK1, while the C-terminus of NRXN3 was able to co-precipitate RSK1 (Fig. S4C). In addition, we investigated the influence of RSK1 on caspase-3 expression. Overexpression of RSK1 led to the decrease of caspase-3 protein, which could be rescued by NRXN3 overexpression (Fig. 3F). On the other hand, we investigated the functional role of targeting RSK1 and found that the RSK1 inhibitor BI-D1870 enhanced the gemcitabine-induced pyroptotic LDH release and ICC sensitivity to gemcitabine (Fig. S4D-E). These results supported the interaction of RSK1 with NRXN3 or caspase-3 and suggested that NRXN3 mediated caspase-3 expression in an RSK1-dependent manner.

Based on the evidence above, we hypothesized that NRXN3 might regulate caspase-3 protein through competing with caspase-3 for binding to RSK1. We first detected if NRXN3 could disrupt the interaction between endogenous caspase-3 and RSK1 in ICC cells. Indeed, NRXN3 overexpression disrupted the co-precipitation of caspase-3 with RSK1 while NRXN3 knockout enhanced the interaction (Fig. 3G and S4F). Then we mixed His-caspase-3- and Myc-RSK1-expressing HEK-293 T lysates in vitro, together with increasing amounts of Flag-NRXN3-expressing lysates. Immunoblotting analysis of anti-Myc IP products revealed that the increasing amounts of Flag-NRXN3-expressing lysates decreased the exogenous interaction between caspase-3 and RSK1 (Fig. 3H). In the same way, the increasing amounts of His-caspase-3-expressing lysates decreased the exogeneous interaction between NRXN3 and RSK1 (Fig. S4G). With the results above consistent with our competitive binding hypothesis, we further performed RSK1 deletion mapping experiments to identify the RSK1 region that interact with NRXN3 or caspase-3. HEK-293 T cells were co-transfected with Flag-NRXN3 and His-caspase-3 along with Myc-tagged full-length RSK1 or deletion mutants. Co-IP assays revealed that deletion of N-terminal kinase (NTK) domain, a domain in RSK1 with kinase catalytic activity, failed to co-precipitate NRXN3 or caspase-3, indicating that both NRXN3 and caspase-3 interacted with the NTK domain of RSK1 and providing deep insight into the structural basis of the competitive binding relationship (Fig. 3I-J).

Overall, our results suggested that NRXN3 competitively blocked caspase-3 interaction with RSK1, thereby regulating caspase-3 protein level and mediating gemcitabine-induced pyroptosis in ICC.

### NRXN3 regulates caspase-3 via RSK1-induced phosphorylation of caspase-3 at T152

**Fig. 4:**
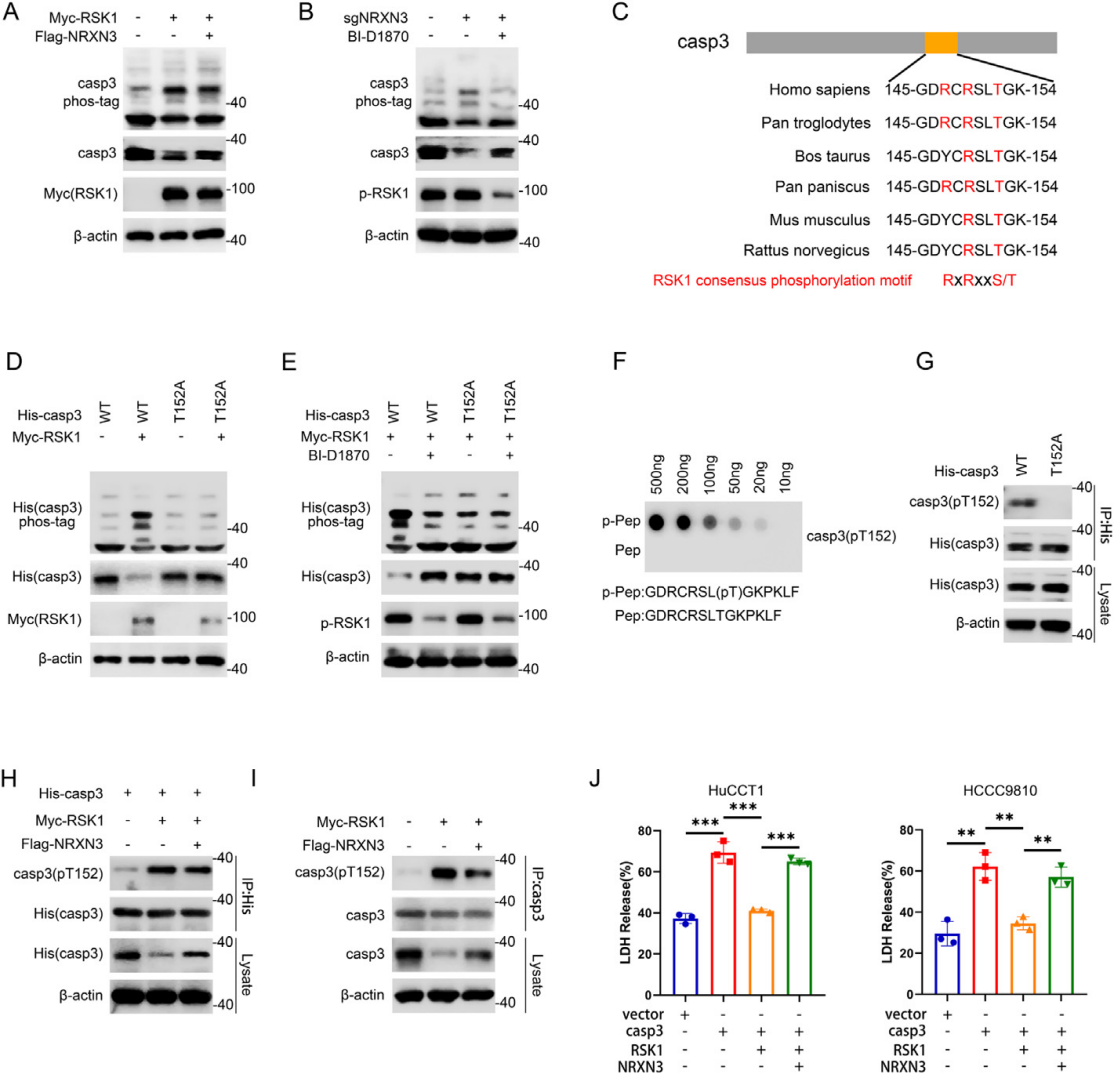
**NRXN3 regulates caspase-3 via RSK1-induced phosphorylation of caspase-3 at T152.** (A) HEK-293 T cells were co-transfected with Myc-RSK1 and Flag-NRXN3 or vector, followed by phos-tag SDS-PAGE and IB. (B) HuCCT1 cells transduced with vector or sgNRXN3 lentiviruses were treated with BI-D1870 (10 μM) for 4 h. Lysates were subjected to phos-tag SDS-PAGE and IB. (C) Alignment of amino acids corresponding to the R-x-R-x-x-S/T sequence across different species with caspase-3. R: arginine, S: serine, T, threonine, x: any residue. (D) HEK-293 T cells were co-transfected with His-caspase-3 wt or His-caspase-3 T152A mutant and Myc-RSK1 or vector, followed by phos-tag SDS-PAGE and IB. (E) HEK-293 T cells were transfected with His-caspase-3 wt or His-caspase-3 T152A mutant and Myc-RSK1, followed by treatment with BI-D1870 as indicated. Lysates were subjected to phos-tag SDS-PAGE and IB. (F) Antibody specificity detection of anti-caspase-3(pT152) antibody. Dot-spot assays were carried out using the anti-caspase-3(pT152) antibody by incubating the phosphorylated or non-phosphorylated peptide containing T152 at the indicated concentration. (G) Antibody specificity detection of anti-caspase-3(pT152) antibody. HEK-293 T cells were transfected with His-caspase-3 wt or His-caspase-3 T152A mutant. Lysates were subjected to anti-His immunoprecipitation and IB as indicated. (H) HEK-293 T cells were co-transfected with His-caspase-3, Myc-RSK1 and Flag-NRXN3 or vector, followed by anti-His immunoprecipitation and IB analysis. (I) HEK-293 T cells were co-transfected with Myc-RSK1 and Flag-NRXN3 or vector, followed by anti-caspase-3 immunoprecipitation and IB analysis. (J) LDH release assays of ICC cells transfected with vector or caspase-3, RSK1 and NRXN3 as indicated in the presence of gemcitabine. *P < 0.05, **P < 0.01, and ***P < 0.001. (J) Student’s *t* test.

Given that RSK1 is a serine/threonine-protein kinase, we detected whether caspase-3 is a phosphorylation substrate of RSK1 to gain a detailed mechanistic insight into how NRXN3 regulates caspase-3 in an RSK1-dependent manner. Kinase assays of caspase-3 protein using Mn^2+^-phos-tag SDS-PAGE suggested that RSK1 promoted the phosphorylation of caspase-3 while NRXN3 attenuated the increased phosphorylation by RSK1 (Fig. 4A). Moreover, knockout of NRXN3 enhanced caspase-3 phosphorylation which was inhibited by the RSK1 inhibitor BI-D1870 (Fig. 4B). The results indicated that RSK1 phosphorylated caspase-3 protein, which was mediated by NRXN3. Previous studies reported that RSK1 phosphorylates substrates on a consensus RxRxxS/T motif [19,20]. Therefore, we performed sequence analysis with ScanProsite tool (https://prosite.expasy.org/scanprosite) and found that residues 147–152 in caspase-3 might be a potential motif for RSK1 phosphorylation. Sequence alignment analysis showed that the threonine at 152 (T152) was highly conserved across different species (Fig. 4C). Based on the clues above, we hypothesized that T152 in caspase-3 might be a putative phosphorylation site by RSK1. To test this hypothesis, we mutated the identified threonine site of caspase-3 protein to alanine (T152A) to evaluate if it was the RSK1 phosphorylation site. Unlike caspase-3 wild-type (WT), RSK1 failed to upregulate the phosphorylation of caspase-3 T152A or downregulate total caspase-3 T152A (Fig. 4D). Besides, BI-D1870 failed to downregulate the phosphorylation of caspase-3 T152A or upregulate total caspase-3 T152A (Fig. 4E). Moreover, caspase-3 T152A exhibited weakened interaction with RSK1 (Fig. S5A). These results indicated that T152 was the indeed phosphorylation site of caspase-3 by RSK1.

To further investigate caspase-3 phosphorylation by RSK1, we developed a polyclonal phospho-specific antibody that exclusively detected T152-phosphorylated caspase-3 (caspase-3 pT152) (Fig. 4F). This antibody was able to detect caspase-3 wt, but not caspase-3 T152A mutant (Fig. 4G). In agreement, the protein level of caspase-3 and caspase-3 pT152 were both reduced in caspase-3-knockdown cells (Fig. S5B). These data suggested that the antibody specifically recognized caspase-3 pT152, thereby we detected caspase-3 pT152 with this antibody in the further study. The results showed that both exogenous and endogenous caspase-3 pT152 protein were upregulated by RSK1 overexpression, whereas further NRXN3 overexpression attenuated the upregulated caspase-3 pT152 (Fig. 4H-I). Besides, knockout of NRXN3 enhanced phosphorylation of exogenous and endogenous caspase-3 at T152, which was compromised upon addition of BI-D1870 (Fig. S5C-D). Functionally, caspase-3 promoted gemcitabine-induced pyroptosis and ICC sensitivity to gemcitabine which were rescued by RSK1, and further NRXN3 overexpression reversed the effects of RSK1 (Fig. 4J and S5E). Together, the results above suggested that RSK1 regulated caspase-3 protein level via phosphorylating caspase-3 at T152, which was mediated by NRXN3.

### RSK1-induced caspase-3 phosphorylation at T152 is recognized by FBXO1 for ubiquitination and degradation

**Fig. 5:**
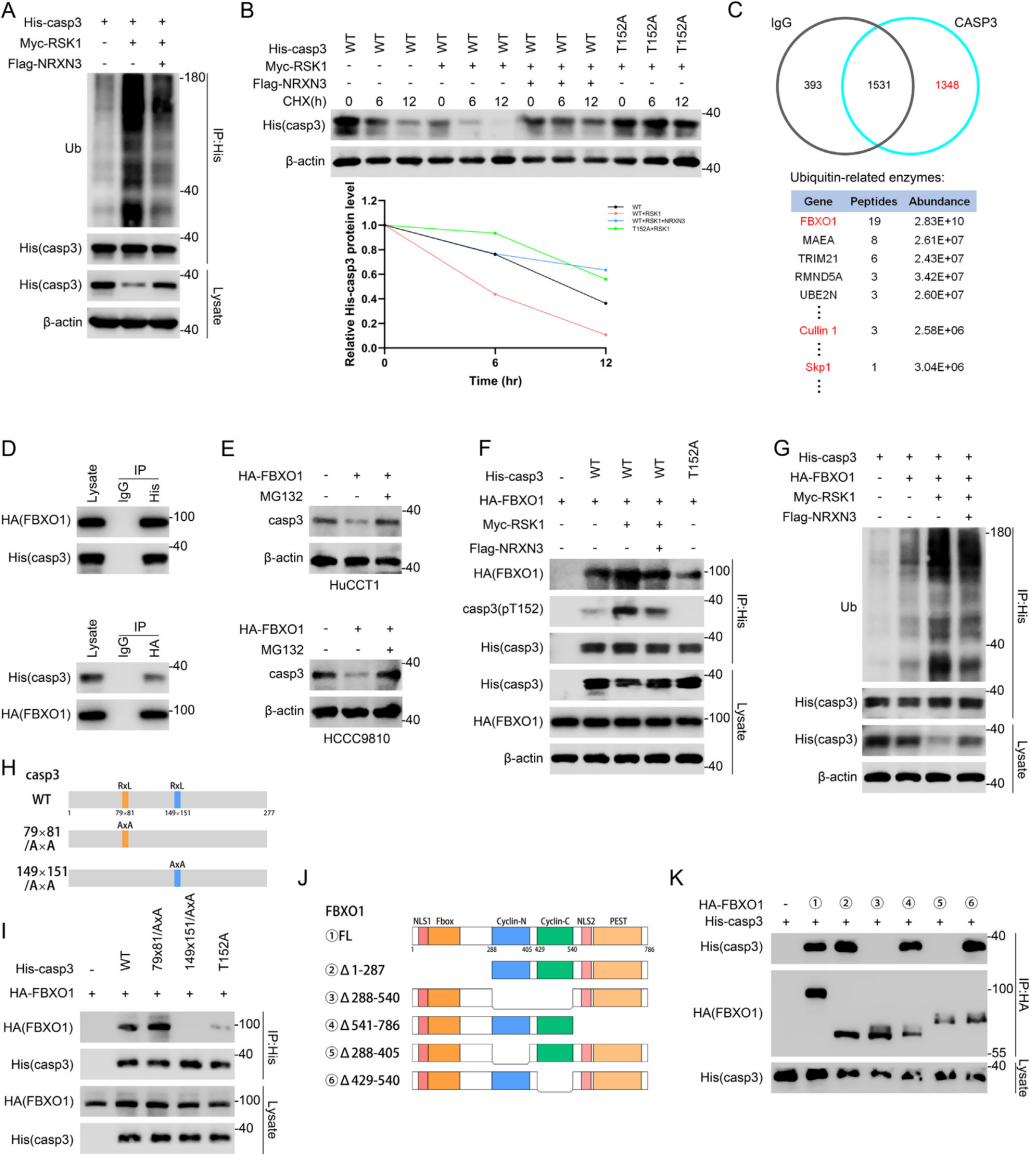
**RSK1-induced caspase-3 phosphorylation at T152 is recognized by FBXO1 for ubiquitination and degradation.** (A) HEK-293 T cells were co-transfected with His-caspase-3, Myc-RSK1 and Flag-NRXN3 or vector, followed by anti-His immunoprecipitation and IB analysis. (B) HEK-293 T cells were transfected as indicated, followed by cycloheximide (CHX, 10 μg/mL) for 0–12 h. Lysates were used for IB to measure the protein levels of caspase-3. Density of caspase-3 expression was quantified by ImageJ and the relative expression compared with the untreated condition is plotted. (C) Data from IP-MS using anti-caspase-3 antibody and normal IgG was analyzed to screen potential ubiquitin ligases or deubiquitinases. Unique peptide numbers and abundance of FBXO1, Cullin 1 and Skp1 in caspase-3 IP-MS is shown. (D) HEK-293 T cells were co-transfected with HA-FBXO1 and His-caspase-3 for 48 h. Cell lysates were analyzed by IP and IB as indicated. (E) HuCCT1 and HCCC9810 cells were transfected with HA-FBXO1 or vector, followed by treatment with DMSO or MG132 (10 μM) for 8 h. (F) HEK-293 T cells were transfected as indicated, followed by anti-His immunoprecipitation and IB analysis. (G) HEK-293 T cells transfected as indicated were subjected to anti-His immunoprecipitation and IB. (H) Schematic representation of caspase-3 wt and caspase-3 mutants highlighting putative FBXO1 binding motifs in caspase-3. (I) HEK-293 T cells were co-transfected with HA-FBXO1 and vector or the indicated His-tagged caspase-3 plasmids. Lysates were subjected to anti-His immunoprecipitation and IB. (J) Schematic representation of full length or various truncated FBXO1. (K) HEK-293 T cells were co-transfected with His-caspase-3 and vector or the indicated HA-tagged FBXO1 plasmids. Lysates were subjected to anti-HA immunoprecipitation and IB.

Following the evidence above, we continued to explore how phosphorylation of caspase-3 by RSK1 could regulate caspase-3 protein level. Since most short-lived proteins are degraded by the ubiquitin–proteasome system, we detected the ubiquitination level of caspase-3 and found that caspase-3 ubiquitination was increased by RSK1 and partly decreased by NRXN3 (Fig. 5A). Knockout of NRXN3 increased the ubiquitination level of caspase-3 while BI-D1870 treatment was able to compromise (Fig. S6A). Cycloheximide (CHX) was used to inhibit protein synthesis to assess the effects of RSK1 and NRXN3 on caspase-3 degradation. As shown in Fig. 5B, RSK1 accelerated the degradation of caspase-3 wt but not caspase-3 T152A, and NRXN3 attenuated the degradation of caspase-3 wt Furthermore, in NRXN3-knockout cells, caspase-3 wt decayed faster while caspase-3 T152A did not exhibit faster degradation. And BI-D1870 treatment rescued the accelerated degradation of caspase-3 wt (Fig. S6B). To gain further insight into the ubiquitination of caspase-3, we examined whether any ubiquitin ligases or deubiquitinating enzymes were identified in our IP-MS analysis. Leave alone the proteins co-precipitated by normal IgG, FBXO1 ranked top according to abundance and unique peptide number among these ubiquitin-related proteins co-precipitated by anti-caspase-3 antibody. FBXO1, also known as CCNF, is the substrate recognition component of a SCF (Skp1-Cullin 1-F box protein) E3 ubiquitin ligase complex. The other two components of the SCF complex, Skp1 and Cullin 1, were also found in the IP-MS analysis (Fig. 5C). The following Co-IP assays verified the exogenous and endogenous FBXO1 interaction with caspase-3 (Fig. 5D and S6C). The immunofluorescence staining demonstrated that FBXO1 localized in both cytoplasm and nucleus, providing spatial foundation for its interaction with the cytoplasm-localized caspase-3 (Fig. S6D). Then we detected the effects of FBXO1 on the stability of caspase-3 protein in ICC cells and the results showed that the stability of caspase-3 was decreased by FBXO1 overexpression, and MG132 (proteasome inhibitor) was able to compromise the changes of caspase-3 stability (Fig. 5E).

Moreover, we investigated if the phosphorylation of caspase-3 at T152 could affect the interaction between caspase-3 and FBXO1. As shown in Fig. 5F, FBXO1 co-precipitated by caspase-3 was upregulated by RSK1 and rescued by additional NRXN3 overexpression, in parallel with the changes of caspase-3 pT152 level. And the interaction with FBXO1 was strongly attenuated by the non-phosphorylatable caspase-3 T152A mutant. Besides, BI-D1870 was able to reverse the enhanced caspase-3 interaction with FBXO1 by NRXN3 knockout (Fig. S6E). Then we explore how the ubiquitination level of caspase-3 was mediated by NRXN3/RSK1/FBXO1 axis. The results showed that FBXO1 increased caspase-3 ubiquitination and decreased caspase-3 stability (Fig. 5G and S6F). RSK1 further enhanced the ubiquitination and decreased the stability of caspase-3, and both were partly reversed by NRXN3 (Fig. 5G). Consistently, NRXN3 knockout promoted the ubiquitination of caspase-3 and inhibited the stability of caspase-3, which were also reversed by BI-D1870 (Fig. S6F). These data indicated that the FBXO1-induced ubiquitination of caspase-3 was subject to NRXN3/RSK1-mediated phosphorylation of caspase-3 at T152.

Subsequently, we explored the binding sites of caspase-3 and FBXO1 to deepen our understanding of the interaction. Previous studies reported that FBXO1 binds to CY motifs (RxL or RxI) of the substrates [21,22]. By mapping the CY motif in caspase-3 sequence, we found that residues 79–81 and 149–151 might be potential FBXO1 docking site. The mutant (RxL/AxA) in the second motif failed to co-precipitate FBXO1, indicating that the CY motif at residues 149–151 of caspase-3 mediates binding to FBXO1. Also, the caspase-3 T152A mutant co-precipitated much less FBXO1, further indicating that phosphorylation at T152 is necessary for an efficient binding to FBXO1 (Fig. 5H-I). In parallel, we constructed multiple FBXO1 deletion mutants and narrowed the caspase-3 binding site to the Cyclin-N domain of FBXO1 (Fig. 5J-K). Together, caspase-3 is a bona fide substrate of FBXO1 for ubiquitination and degradation, and phosphorylation at T152 is necessary for caspase-3 binding to FBXO1.

### NRXN3 regulates gemcitabine-induced pyroptosis through RSK1/FBXO1-mediated caspase-3 degradation

**Fig. 6:**
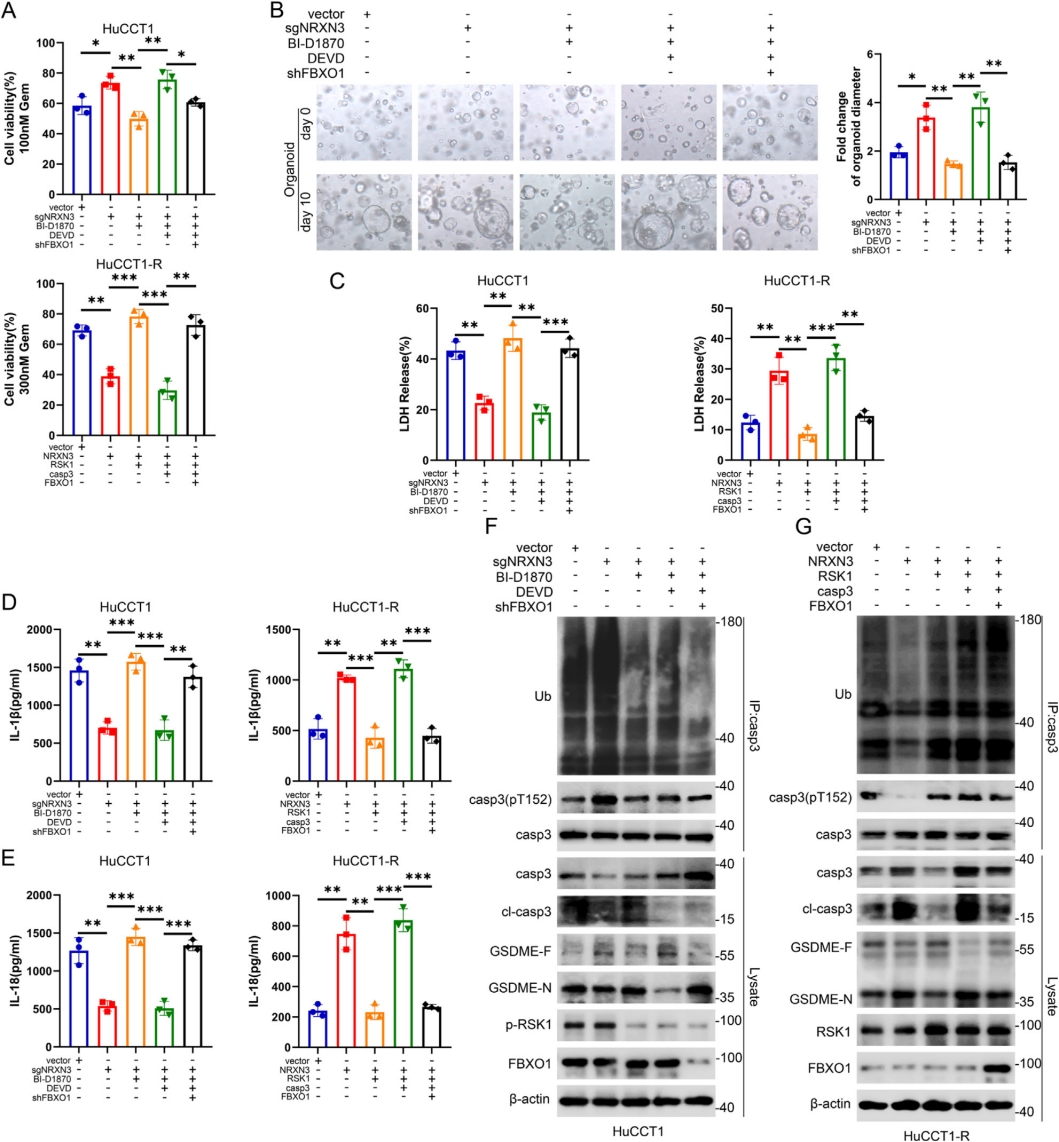
**NRXN3 regulates gemcitabine-induced pyroptosis through RSK1/FBXO1-mediated caspase-3 degradation.** (A) Cell viability of ICC cells treated with gemcitabine at the indicated dose for 48 h. HuCCT1 cells transduced with vector or sgNRXN3 and shFBXO1 lentiviruses were treated with BI-D1870 and AC-DEVD-CHO (DEVD, 100 μM) as indicated. HuCCT1-R cells transduced with vector or NRXN3-overexpressing lentiviruses were co-transfected with RSK1, caspase-3 and FBXO1 as indicated. (B) Representative images and diameter fold change of the patient-derived organoid at day 0 and day 10 after treatment with gemcitabine. The organoid transduced with vector or sgNRXN3 and shFBXO1 lentiviruses were treated as indicated in the presence of gemcitabine. (C) HuCCT1 and HuCCT1-R cells were treated as in (A). LDH release assays were conducted after exposure to gemcitabine (5000 nM) for 48 h. (D) HuCCT1 and HuCCT1-R cells were treated as in (A). Measurement of IL-1β release was conducted after exposure to gemcitabine (5000 nM) for 48 h. (E) HuCCT1 and HuCCT1-R cells were treated as in (A). Measurement of IL-18 release was conducted after exposure to gemcitabine (5000 nM) for 48 h. (F) HuCCT1 treated as in (C) were subjected to anti-caspase-3 immunoprecipitation and IB. (G) HuCCT1-R treated as in (C) were subjected to anti-caspase-3 immunoprecipitation and IB. *P < 0.05, **P < 0.01, and ***P < 0.001. (A-E) Student’s *t* test.

The experiments above demonstrated phospho-dependent ubiquitination and degradation of caspase-3 mediated by NRXN3/RSK1/FBXO1 axis. Next, we would detect the influence of this regulatory axis on gemcitabine-induced pyroptosis in ICC cells. As shown in Fig. 6A-B and S7A, the cell viability, colony formation and migration of HuCCT1 cells and growth of the ICC organoid were enhanced by NRXN3 knockout in the presence of gemcitabine, and BI-D1870 reversed these malignant phenotypes. Further AC-DEVD-CHO (caspase-3 inhibitor) treatment was able to counteract the effects of BI-D1870 treatment and facilitate chemoresistance, which was significantly rescued by knockdown of FBXO1. Moreover, the cell viability, colony formation and migration of HuCCT1-R cells in the presence of gemcitabine, hampered by NRXN3, were rescued by RSK1, which were significantly inhibited by caspase-3 overexpression. Further overexpression of FBXO1 enhanced the downregulated tumorigenesis and migration by caspase-3 (Fig. 6A and S7B). In gemcitabine-treated HuCCT1 cells, release of LDH, IL-1β and IL-18, percentage of pyroptotic cell death and Tunel-positive cells were inhibited by NRXN3 knockout, which were reversely enhanced by BI-D1870 treatment. AC-DEVD-CHO treatment was able to counteract the effects of BI-D1870, which would be reversed by FBXO1 knockdown. Conversely, the enhanced pyroptosis in NRXN3-overexpression HuCCT1-R cells were offset by RSK1. Further caspase-3 overexpression could boost the pyroptotic effects which could be inhibited by FBXO1 (Fig. 6C-E, S7C-D). As shown in Fig. 6F-G, NRXN3 knockout increased the phosphorylated caspase-3 at T152 along with the ubiquitination level and facilitated the degradation of caspase-3, thus downregulating the cleavage of full-length GSDME. Conversely, NRXN3 overexpression decreased phospho-T152 and ubiquitination level of caspase-3, upregulating caspase-3 protein level and the downstream cleavage of GSDME. We observed that BI-D1870 treatment was able to counteract the effects of NRXN3 knockout while RSK1 overexpression was able to offset the effects of NRXN3 overexpression. AC-DEVD-CHO treatment was able to inhibited the cleavage of caspase-3 and thus inhibited the cleavage of GSDME. FBXO1 knockdown upregulated caspase-3 protein level and partly compromised the effects of AC-DEVD-CHO. On the contrary, overexpression of caspase-3 significantly promoted the cleavage of GSDME, but FBXO1 overexpression led to the degradation of caspase-3 and rescued the cleavage of GSDME. Overall, the data above indicated NRXN3 facilitated gemcitabine-induced GSDME-dependent pyroptosis in ICC through inhibiting RSK1/FBXO1-mediated phosphorylation and degradation of caspase-3 protein.

### NRXN3/RSK1/FBXO1 axis regulates in vivo chemotherapy response through mediating caspase-3/GSDME pathway in ICC

**Fig. 7:**
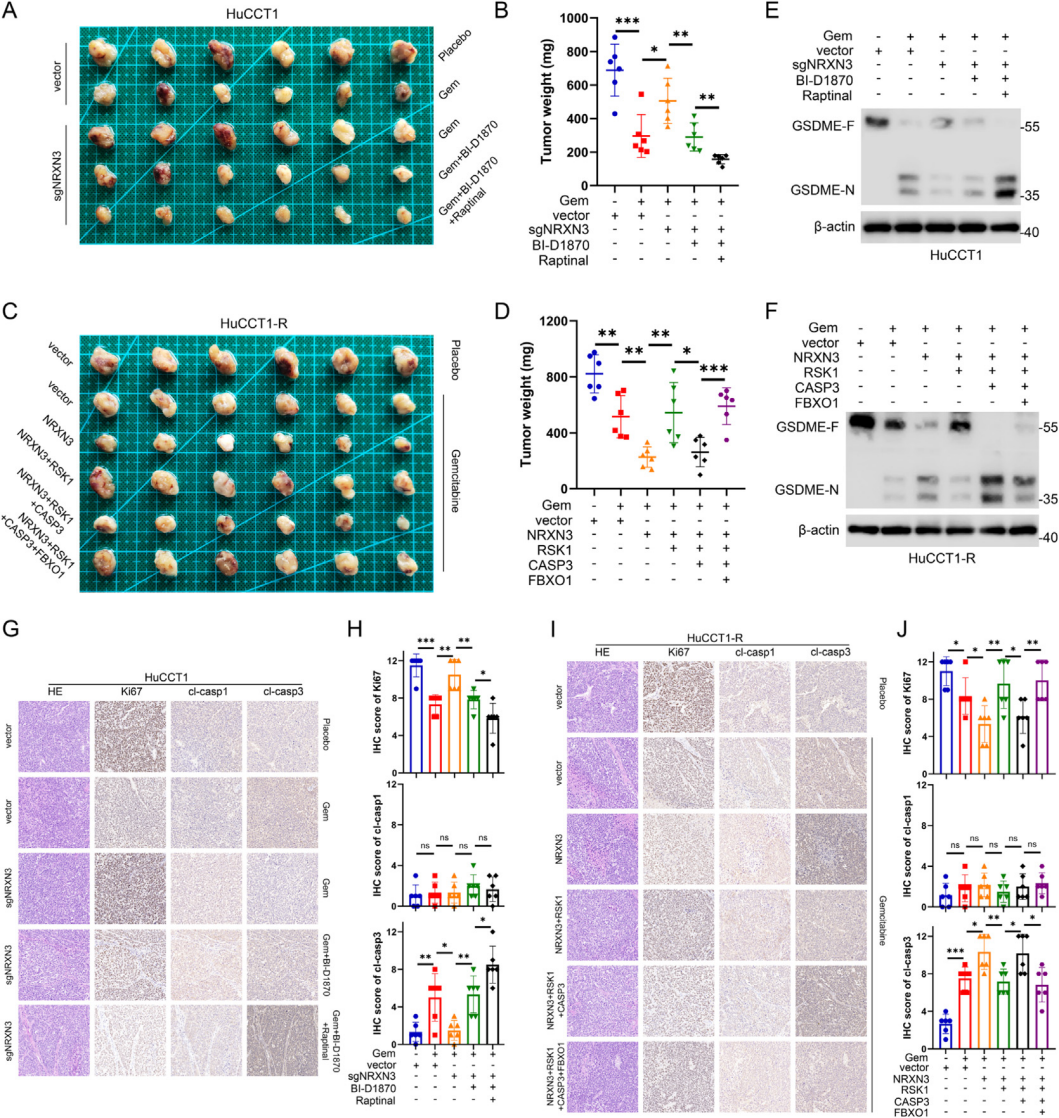
**NRXN3/RSK1/FBXO1 axis regulates in vivo chemotherapy response through mediating caspase-3/GSDME pathway in ICC.** (A) Xenograft tumors in nude mice generated with HuCCT1 cells. The cells were genetically modified and treated as indicated. (B) Scatter chart represents the tumor weight of each group in (A). (C) Xenograft tumors in nude mice generated with HuCCT1-R cells. The cells were genetically modified as indicated. (D) Scatter chart represents the tumor weight of each group in (C). (E) Immunoblotting analysis of GSDME in xenograft tumors from each group in (A). (F) Immunoblotting analysis of GSDME in xenograft tumors from each group in (C). (G) Representative IHC staining images with the indicated antibodies of HuCCT1 xenograft tumor serial sections from each group in (A). (H) IHC score of Ki67, cl-casp1 and cl-casp3 in xenograft tumors from each group in (A). (I) Representative IHC staining images with the indicated antibodies of HuCCT1-R xenograft tumor serial sections from each group in (C). (J) IHC score of Ki67, cl-casp1 and cl-casp3 in xenograft tumors from each group in (C). ^ns^P > 0.05, *P < 0.05, **P < 0.01, and ***P < 0.001. (B, D, H, J) Student’s *t* test.

We performed in vivo experiments to evaluate the regulatory effects of NRXN3/RSK1/FBXO1/caspase-3 axis on ICC chemotherapy responses. The genetically modified HuCCT1 or HuCCT1-R cells were injected subcutaneously into nude male mice, which were later subjected to different agents. The results showed that gemcitabine treatment suppressed tumor growth in vivo, especially in the sensitive HuCCT1 xenografts. In HuCCT1-developed xenografts, knockout of NRXN3 reversed the antitumor effects of gemcitabine, but BI-D1870 inhibited the tumor growth caused by NRXN3 knockout. Raptinal treatment synergized with BI-D1870 to enhance chemosensitivity and suppress tumor growth (Fig. 7A-B). In HuCCT1-R-developed xenografts, NRXN3 overexpression sensitized tumors to gemcitabine and further inhibited tumor growth, while RSK1 overexpression reversely promoted tumor growth. Overexpression of caspase-3 rescued the sensitivity of tumors to gemcitabine and significantly inhibited tumor growth, which could be offset by FBXO1 overexpression (Fig. 7C-D). In addition, we performed immunoblotting analysis and immunohistochemical staining of tumors from each treatment group. Immunoblotting analysis showed that gemcitabine treatment promoted the cleavage of GSDME which changed in consistence with the tumor sensitivity to gemcitabine (Fig. 7E-F). Immunohistochemical staining revealed that Ki67 staining changed in tune with tumor size of each group while expression of cleaved-caspase-3 showed significantly opposite changes with tumor growth and chemoresistance. Conversely, no notable difference in cleaved-caspase-1 staining was observed (Fig. 7G-J). Collectively, these results suggested that NRXN3/RSK1/FBXO1 axis regulated in vivo chemotherapy response through mediating caspase-3/GSDME pathway in ICC, and disruption of this regulatory axis could promote chemosensitivity or confer chemoresistance. Pharmaceutical inhibition of RSK1 or activation of caspase-3 could be an improved therapeutic strategy for ICC.

## Discussion

ICC remains one of the most lethal malignancies for patients, and the majority of patients present with unresectable disease with systemic therapy being the only treatment available. Standard chemotherapy for patients with ICC is gemcitabine-based treatment. However, the therapeutic efficacy of gemcitabine is frequently limited due to development of chemoresistance [1,2,23]. Accumulating evidence has shown that cancer cells often develop resistance to chemotherapy by impairing or bypassing apoptotic cell death [[24], [25], [26], [27], [28]]. The dilemma demonstrates the importance of strategies targeting non-apoptotic cell death. Pyroptosis, a programmed cell death associated with inflammatory response, is triggered by inflammasomes and executed by the caspase and gasdermin protein family[3,4]. Pyroptosis was originally found to initiate innate immunity by the release of cytokines and other molecules after cell membrane rupture. Intriguingly, emerging evidence has proven that pyroptosis plays a crucial role in cancer progression and response to chemotherapy [29]. Recent studies have shown that chemotherapeutic agents can induce pyroptotic cell death in multiple malignancies through cleavage of GSDME by caspase-3, highlighting the significance of caspase-3/GSDME pyroptotic pathway in chemotherapy response [[5], [6], [7], [8], [9]]. However, the mechanism of pyroptosis has not yet been thoroughly elucidated, and the role of pyroptosis in ICC treatment has been rarely studied. In this study, we investigated the role of pyroptosis in ICC in search of strategies to improve the response to currently available therapies. Our findings demonstrated that gemcitabine treatment was able to trigger robust pyroptosis in ICC cells. Concordant with the previous studies, the present study attaches great importance to the role of caspase-3/GSDME pathway in chemotherapy.

Caspase-3, a protease that acts as a major effector caspase in both apoptosis and pyroptosis, is a promising therapeutic target in cancer treatment. Caspase-3 is an important biomarker for the evaluation of chemotherapy response and increasing evidence has shown that altering caspase-3 expression or activity can confer therapeutic benefits [30]. Previous studies have shown that the stability and activity of caspase-3 are modulated by multiple post-translational modifications (PTMs), such as phosphorylation, ubiquitination, nitrosylation, and glutathionylation [31]. Previous studies have reported both proapoptotic and antiapoptotic effects of caspase-3 phosphorylation. The activity of caspase-3 is reported to be inhibited in neutrophils by p38-MAPK via phosphorylation at S150, which is reversed by protein phosphatase 2A via dephosphorylation [32,33]. Nevertheless, PKCδ phosphorylates caspase-3 at S26 and enhances caspase-3 activity in monocytes [34]. Researchers found that SCF(β-TrCP) promotes ubiquitination of caspase-3 at the N-terminal domain and its further degradation, which is counteracted by ubiquitin-specific protease 15 (USP15) via deubiquitinating caspase-3 and thereby increasing its stability [35,36]. Inhibitors of apoptotic proteins (IAPs) that have a RING finger domain with ubiquitin ligase activity can also promote degradation of caspase-3 while some other deubiquitinating enzymes, such as USP41 or ataxin-2, confer activation of caspase-3 [[37], [38], [39]]. Nitrosylation and glutathionylation have also been reported to modulate caspase-3 activity via diverse mechanisms [[40], [41], [42], [43]]. As mentioned above, caspase-3 stability and activity are regulated by various PTMs. But few studies explored the effects of crosstalk between PTMs on the stability and function of caspase-3. In the present study, we demonstrated novel phosphorylation and ubiquitination modification of caspase-3, and took a deep dive into the crosstalk between RSK1-dependent phosphorylation and FBXO1-dependent ubiquitination of caspase-3. Through IP-MS and Co-IP, we identified that caspase-3 was a bona fide substrate of RSK1 for phosphorylation and of FBXO1 for ubiquitination. Phosphorylation of caspase-3 at T152 by RSK1 promoted caspase-3 binding to FBXO1 for ubiquitination and degradation, thereby dampening GSDME-dependent pyroptotic cell death in ICC. Specific RSK1 inhibitor BI-D1870 suppressed kinase activity of RSK1 and attenuated phosphorylation and degradation of caspase-3 to exacerbate pyroptosis. Our results deepen our understanding of the modulation of caspase-3 stability, and pave the way to novel pyroptosis-based therapies for ICC.

The gene NRXN3 has been frequently studied in the context of neurological functions. Recently, some studies revealed that NRXN3 plays a crucial role in the progression of several cancer types. Sun et al. and Liu et al. found that NRXN3 could inhibit glioma proliferation, migration and invasion [13,14]. Zhao et al. reported the tumor-suppressor role of NRXN3 in nasopharyngeal carcinoma [15]. However, the function of NRXN3 in ICC, chemotherapy and pyroptosis has not yet been studied. Herein, through integrating genome-scale CRISPR-Cas9 screen with transcriptomic analysis, NRXN3 was identified as a key contributor to pyroptosis and gemcitabine sensitivity in ICC. The analysis of our ICC patient cohort revealed that NRXN3 expression positively correlated with prognosis of ICC patients. Moreover, we found that NRXN3 facilitated pyroptosis and chemosensitivity in ICC. Through a series of assays, we identified that NRXN3 competitively bound to the NTK domain of RSK1 and blocked RSK1 interaction with caspase-3, thereby regulating caspase-3 stability and downstream GSDME-dependent pyroptosis. Further experiments demonstrated the in-depth mechanisms of NRXN3 regulating caspase-3 stability in a phospho- and ubiquitin-dependent manner mediated by RSK1 and FBXO1. The results pinpoint the therapeutic strategy of blocking the NTK domain of RSK1 by specific antibody in the modulation of pyroptosis. Our study is the first to discover the significance of NRXN3 in chemotherapy-induced pyroptosis and elucidate the mechanisms underneath the phenotype, highlighting the potential of NRXN3 as the biomarker to predict chemotherapy response and prognosis for patients with ICC. Through survival analysis with Kaplan-Meier Plotter database, we also observed the broad involvement of NRXN3 in other malignancies treated with various therapies such as sorafenib-treated liver cancer, implying the universal significance of NRXN3 across a broader spectrum of cancer types. However, this type of analysis alone is inadequate for extrapolating the conclusions derived from ICC to other malignancies. Further quantitative studies are needed to validate the role of NRXN3 and the regulatory mechanism of NRXN3/RSK1/FBXO1/caspase-3 axis in other types of cancer.

## Conclusion

The present study revealed that the NRXN3/RSK1/FBXO1-mediated stability of caspase-3 regulated gemcitabine-induced pyroptosis in ICC. In addition, administration of an RSK1 inhibitor or caspase-3 activator, such as BI-D1870 or Raptinal, facilitated ICC response to gemcitabine by activating pyroptosis. Our study helps to gain insight into the modulation of pyroptosis and sheds light on new strategies for overcoming chemoresistance and improving survival.

## Compliance with ethics requirements

*Studies with human specimens were approved by the Ethics Committee of the First Affiliated Hospital of Nanjing Medical University (2019-SR-133), and written informed patient consent was obtained in accordance with regional regulations.*

*Animal experiments were approved by the Institutional Animal Care and Use Committee of Nanjing Medical University* (IACUC-2304016) *and conducted according to the criteria outlined in the “Guide for the Care and Use of Laboratory Animals”.*

## CRediT authorship contribution statement

**Tao Zhou:** Conceptualization, Investigation, Data curation, Formal analysis, Visualization, Writing – original draft, Writing – review & editing. **Yaodong Zhang:** Conceptualization, Writing – review & editing, Funding acquisition, Project administration. **Tianlin Wang:** Data curation, Formal analysis, Visualization, Resources. **Jiang Chang:** Investigation, Data curation, Formal analysis, Visualization, Writing – review & editing. **Wangjie Jiang:** Investigation, Data curation, Formal analysis, Visualization, Writing – review & editing. **Yananlan Chen:** Data curation, Formal analysis, Visualization. **Shenye Shao:** Resources. **Ruixiang Chen:** Resources. **Jifei Wang:** Resources. **Yirui Wang:** Investigation. **Changxian Li:** Writing – review & editing, Project administration. **Xiangcheng Li:** Writing – review & editing, Funding acquisition, Project administration.

## Funding

This study was supported by the National Natural Science Foundation of China (82472865, 82273066), Natural Science Foundation of Jiangsu Province (BK20220723), and Major Basic Research Fund of Jiangsu Province Hospital (QY202404).

## Declaration of competing interest

*The authors declare that they have no known competing financial interests or personal relationships that could have appeared to influence the work reported in this paper.*

## Data Availability

The data that support the findings of this study are available from the corresponding author upon reasonable request.
