# Supplementary material for: NRXN3 regulates pyroptosis in intrahepatic cholangiocarcinoma via mediating the phospho-dependent ubiquitination and degradation of caspase-3
Source: J Adv Res. 2025 May 3;80:655–69. doi: 10.1016/j.jare.2025.04.040 (PMC12869240; doi:10.1016/j.jare.2025.04.040)
Supplement: Supplementary Data 1 [file mmc1.docx]

**Supplementary Tables**

**Table. S1 Association of NRXN3 expression with clinicopathological features of ICC.**

| **Clinicopathological features** | **Tumor NRXN3 expression** | | **P value** | **χ2** |
| --- | --- | --- | --- | --- |
|  | **Low** | **High** |  |  |
| **All cases** | 62(51.7%) | 58(48.3%) |  |  |
| **Gender** |  |  | 0.261 | 1.261 |
| Male | 30(48.0%) | 34(59.0%) |  |  |
| Female | 32(52.0%) | 24(41.0%) |  |  |
| **Age** |  |  | 0.296 | 1.090 |
| ≤60 | 40(65.0%) | 32(55.0%) |  |  |
| ＞60 | 22(35.0%) | 26(45.0%) |  |  |
| **CA199(U/L)** |  |  | **0.013*** | 6.108 |
| ≤37 | 5(8.1%) | 9(15.5%) |  |  |
| ＞37 | 21(33.9%) | 7(12.1%) |  |  |
| **CEA(ng/ml)** |  |  | 0.735 | 0.115 |
| ≤5 | 16(25.8%) | 9(15.5%) |  |  |
| ＞5 | 10(16.1%) | 7(12.1%) |  |  |
| **AFP(ng/ml)** |  |  | 0.903 | 0.015 |
| ≤20 | 24(38.7%) | 18(31.0%) |  |  |
| ＞20 | 3(4.8%) | 2(3.5%) |  |  |
| **Diameter(cm)** |  |  | 0.854 | 0.034 |
| ≤3 | 12(19.4%) | 12(20.7%) |  |  |
| ＞3 | 33(53.2%) | 36(62.1%) |  |  |
| **Tumor No.** |  |  | 0.384 | 0.757 |
| 1 | 15(24.2%) | 16(27.6%) |  |  |
| ≥2 | 7(11.3%) | 4(6.9%) |  |  |
| **Histological grade** |  |  | 0.665 | 0.188 |
| I/I-II/II | 23(37.1%) | 20(34.5%) |  |  |
| II-III/III | 32(51.6%) | 33(56.9%) |  |  |
| **Nerve invasion** |  |  | 0.525 | 0.404 |
| Absent | 33(53.2%) | 38(65.5%) |  |  |
| Present | 15(24.2%) | 13(22.4%) |  |  |
| **Tumor thrombus** |  |  | 0.776 | 0.081 |
| Absent | 49(79.0%) | 47(81.0%) |  |  |
| Present | 10(16.1%) | 11(19.0%) |  |  |
| **T stage** |  |  | 0.377 | 0.780 |
| Tis-T2 | 56(90.3%) | 52(89.7%) |  |  |
| T3-T4 | 2(3.2%) | 4(6.9%) |  |  |
| **N stage** |  |  | 0.200 | 1.643 |
| N0 | 43(69.4%) | 47(81.0%) |  |  |
| N1, N2 | 15(24.2%) | 9(15.5%) |  |  |
| **M stage** |  |  | 0.161 | 1.966 |
| M0 | 56(90.3%) | 56(96.6%) |  |  |
| M1 | 2(3.2%) | 0(0.0%) |  |  |
| **Clinical stage** |  |  | 0.244 | 1.356 |
| I/II | 40(64.5%) | 44(75.9%) |  |  |
| III/IV | 18(29.0%) | 12(20.7%) |  |  |
| **Surgical margin** |  |  | 0.797 | 0.066 |
| R0 | 54(87.1%) | 53(91.4%) |  |  |
| R1，R2 | 6(9.7%) | 5(8.6%) |  |  |
| **Recurrence** |  |  | **0.028*** | 4.822 |
| Absent | 15(24.2%) | 25(43.1%) |  |  |
| Present | 47(75.8%) | 33(56.9%) |  |  |

**Table. S2 Antibodies and reagents.**

| **Antibody or reagent** | **Source** | **Identifier** |
| --- | --- | --- |
| rabbit polyclonal anti-NRXN3 | Affinity, Jiangsu, China | DF9682 |
| rabbit polyclonal anti-β-actin | Proteintech, Hubei, China | 20536-1-AP |
| rabbit monoclonal anti-NLRP3 | Abcam, Cambridge, UK | ab270449 |
| rabbit polyclonal anti-caspase-1 | Proteintech, Hubei, China | 22915-1-AP |
| rabbit polyclonal anti-cleaved-caspase-1 | Thermofisher, Waltham, USA | PA5-38099 |
| rabbit monoclonal anti-GSDMD | Abcam, Cambridge, UK | ab209845 |
| rabbit monoclonal anti-caspase-3 | Abcam, Cambridge, UK | ab32351 |
| mouse monoclonal anti-caspase-3 | Proteintech, Hubei, China | 66470-2-Ig |
| rabbit polyclonal anti-cleaved-caspase-3 | Cell signaling, Massachusetts, USA | #9661 |
| rabbit monoclonal anti-GSDME | Abcam, Cambridge, UK | ab215191 |
| rabbit polyclonal anti-Flag | Proteintech, Hubei, China | 20543-1-AP |
| mouse monoclonal anti-Flag | Cell signaling, Massachusetts, USA | #8146 |
| rabbit polyclonal anti-Myc | Proteintech, Hubei, China | 16286-1-AP |
| mouse monoclonal anti-Myc | Cell signaling, Massachusetts, USA | #2276 |
| rabbit polyclonal anti-His | Proteintech, Hubei, China | 10001-0-AP |
| mouse monoclonal anti-His | Abcam, Cambridge, UK | ab18184 |
| rabbit polyclonal anti-HA | Proteintech, Hubei, China | 51064-2-AP |
| mouse monoclonal anti-HA | Proteintech, Hubei, China | 66006-2-Ig |
| rabbit monoclonal anti-RSK1 | Abcam, Cambridge,UK | ab32114 |
| rabbit polyclonal anti-RSK1 (phospho-S221) | Abcam, Cambridge, UK | ab10695 |
| rabbit polyclonal anti-caspase-3 (phospho-T152) | Lifetein, Beijing, China | Customized, RRID: AB_3097756 |
| mouse monoclonal anti-Ubiquitin | Abcam, Cambridge, UK | ab7254 |
| rabbit polyclonal anti-FBXO1 | Thermofisher, Waltham, USA | A303-406A |
| Gemcitabine (Gem) | MedChemExpress, New Jersey, USA | HY-17026 |
| BI-D1870 | MedChemExpress, New Jersey, USA | HY-10510 |
| cycloheximide (CHX) | MedChemExpress, New Jersey, USA | HY-12320 |
| MG132 | MedChemExpress, New Jersey, USA | HY-13259 |
| AC-DEVD-CHO (DEVD) | MedChemExpress, New Jersey, USA | HY-P1001 |
| Raptinal | MedChemExpress, New Jersey, USA | HY-121320 |

**Table. S3 Primers, sgRNAs and siRNAs.**

| **Name** | **Sense (5’-3’)** |
| --- | --- |
| NRXN3-Forward | CGCTACGTACATCTTTGGGA |
| NRXN3-Reverse | TGCTGGGCCTGTCATTGG |
| PCDH1-Forward | ACGCCACTCGGGTAGTGTA |
| PCDH1-Reverse | TCACGGTCGATGGAGGTCTC |
| CACNG4-Forward | CATCGAAGGGATCTATAAAGGGC |
| CACNG4-Reverse | GAGGAGGTACTCCGAGCTGT |
| caspase-3-Forward | CATGGAAGCGAATCAATGGACT |
| caspase-3-Reverse | CTGTACCAGACCGAGATGTCA |
| GSDME-Forward | ACATGCAGGTCGAGGAGAAGT |
| GSDME-Reverse | TCAATGACACCGTAGGCAATG |
| β-Actin-Forward | CATGTACGTTGCTATCCAGGC |
| β-Actin-Reverse | CTCCTTAATGTCACGCACGAT |
| sgNRXN3-1 | AGGCGGTCAGACCGCGTGCT |
| sgNRXN3-2 | GCTGGCGCTACGTACATCTT |
| NRXN3-KO-Forward (for sanger seq) | GGACATTTCCTCTCTGCAGCTGTTCTGTG |
| NRXN3-KO-Reverse (for sanger seq) | AAGCTGGAGGAAGTCACCAAGTCCTGG |
| si-NRXN3-Sense | GAGGUUGUUUAUAAGAAUAAU |
| si-NRXN3-Antisense | UAUUCUUAUAAACAACCUCUU |
| si-PCDH1-Sense | AGAUAGAAGUACAAGACAUCA |
| si-PCDH1-Antisense | AUGUCUUGUACUUCUAUCUGG |
| si-CACNG4-Sense | GCAUCGAAGGGAUCUAUAAAG |
| si-CACNG4-Antisense | UUAUAGAUCCCUUCGAUGCAG |
| si-caspase-3#1-Sense | GGUAGUUGCAAUUGAAUUAAA |
| si-caspase-3#1-Antisense | UAAUUCAAUUGCAACUACCUG |
| si-caspase-3#2-Sense | GCGUGAUGUUUCUAAAGAAGA |
| si-caspase-3#2-Antisense | UUCUUUAGAAACAUCACGCAU |
