# Supplementary material for: NRXN3 regulates pyroptosis in intrahepatic cholangiocarcinoma via mediating the phospho-dependent ubiquitination and degradation of caspase-3
Source: J Adv Res. 2025 May 3;80:655–69. doi: 10.1016/j.jare.2025.04.040 (PMC12869240; doi:10.1016/j.jare.2025.04.040)
Supplement: Supplementary Data 2 [file mmc2.docx]

**Supplementary Figures**

**Fig. S1**

**
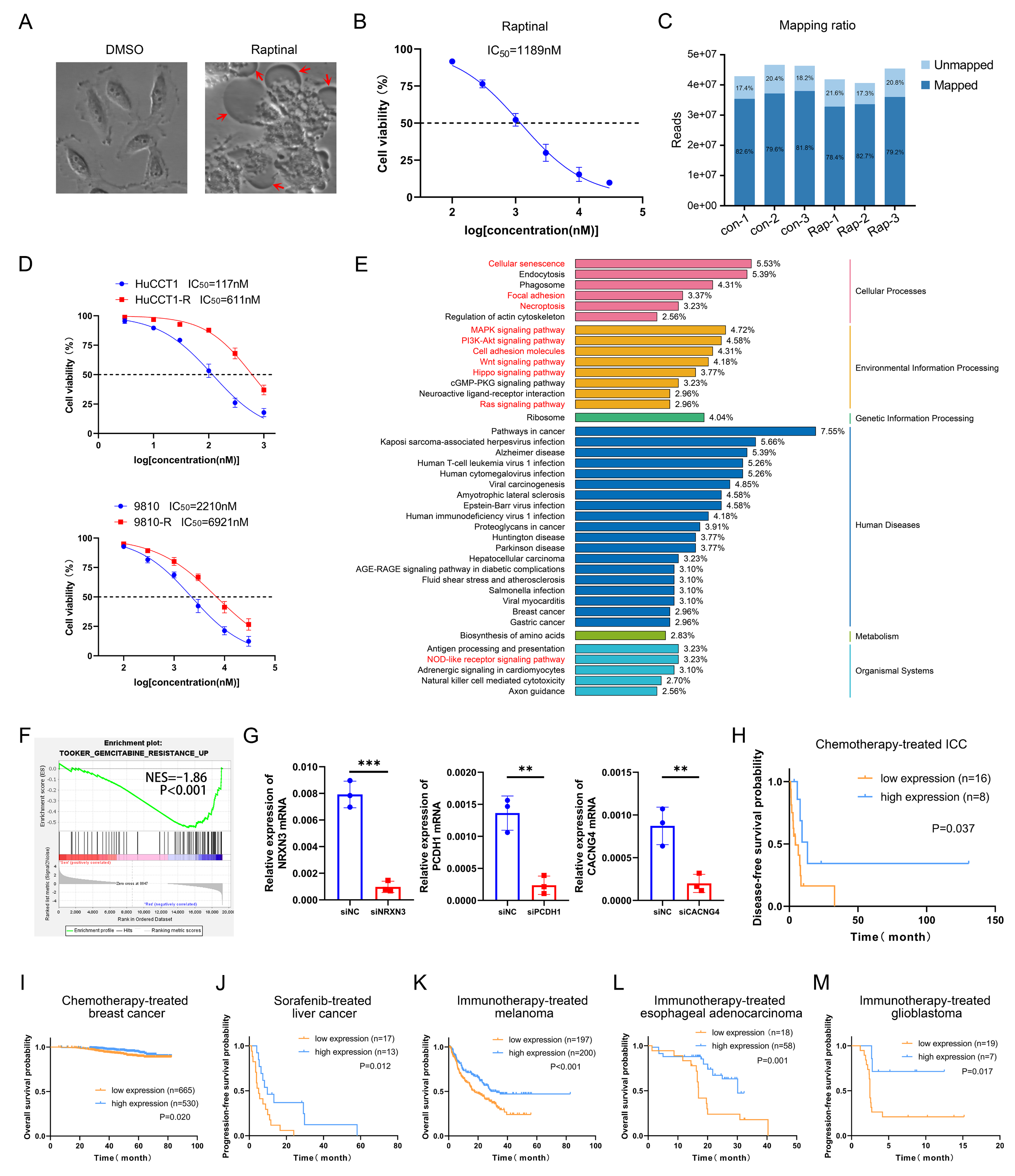
**

**Fig. S1, related to Fig. 1 Integrated whole-genome CRISPR screen with transcriptomic analysis to reveal critical contributors to both pyroptosis and chemosensitivity in ICC.**

(A) Representative high-throughput bright-field images of HuCCT1 cells treated with Raptinal. Red arrows indicate characteristic ballooning in cell membranes. (B) Raptinal IC50 experiments of HuCCT1. (C) Total reads and mapping ratio of the CRISPR screen. (D) Gemcitabine IC50 experiments of HuCCT1, HuCCT1-R, HCCC9810 and HCCC9810-R. (E) KEGG pathway enrichment analysis of the transcriptomic data from HuCCT1 and HuCCT1-R cells revealed an enrichment in some important pathways that are involved in cancer development, chemoresistance and pyroptosis. (F) Gene set enrichment analysis (GSEA) demonstrated that genes associated with upward gemcitabine resistance were significantly enriched in gemcitabine resistant HuCCT1-R, confirming the successful establishment of the resistant cells. (G) RT-qPCR analysis was used to verify the knockdown efficiency of NRXN3, PCDH1 and CACNG4 in HuCCT1 cells. (H) Correlation between NRXN3 expression and disease-free survival in chemotherapy-treated ICC patient. (I) Kaplan-Meier overall survival curves of patients with breast cancer treated with chemotherapy with high or low NRXN3 expression (n=1195, KM plotter database). (J) Correlation between NRXN3 expression and progression-free survival in patient with liver cancer treated with sorafenib (n=30, KM plotter database). (K) Kaplan-Meier overall survival curves of patients with melanoma treated with immunotherapy with high or low NRXN3 expression (n=397, KM plotter database). (L) Kaplan-Meier overall survival curves of patients with esophageal adenocarcinoma treated with immunotherapy with high or low NRXN3 expression (n=76, KM plotter database). (M) Correlation between NRXN3 expression and progression-free survival in patient with glioblastoma treated with immunotherapy (n=26, KM plotter database). *P < 0.05, **P < 0.01, and ***P < 0.001. (G) Student’s t test. (H-M) Log-rank (Mantel-Cox) test.

**Fig. S2**

**
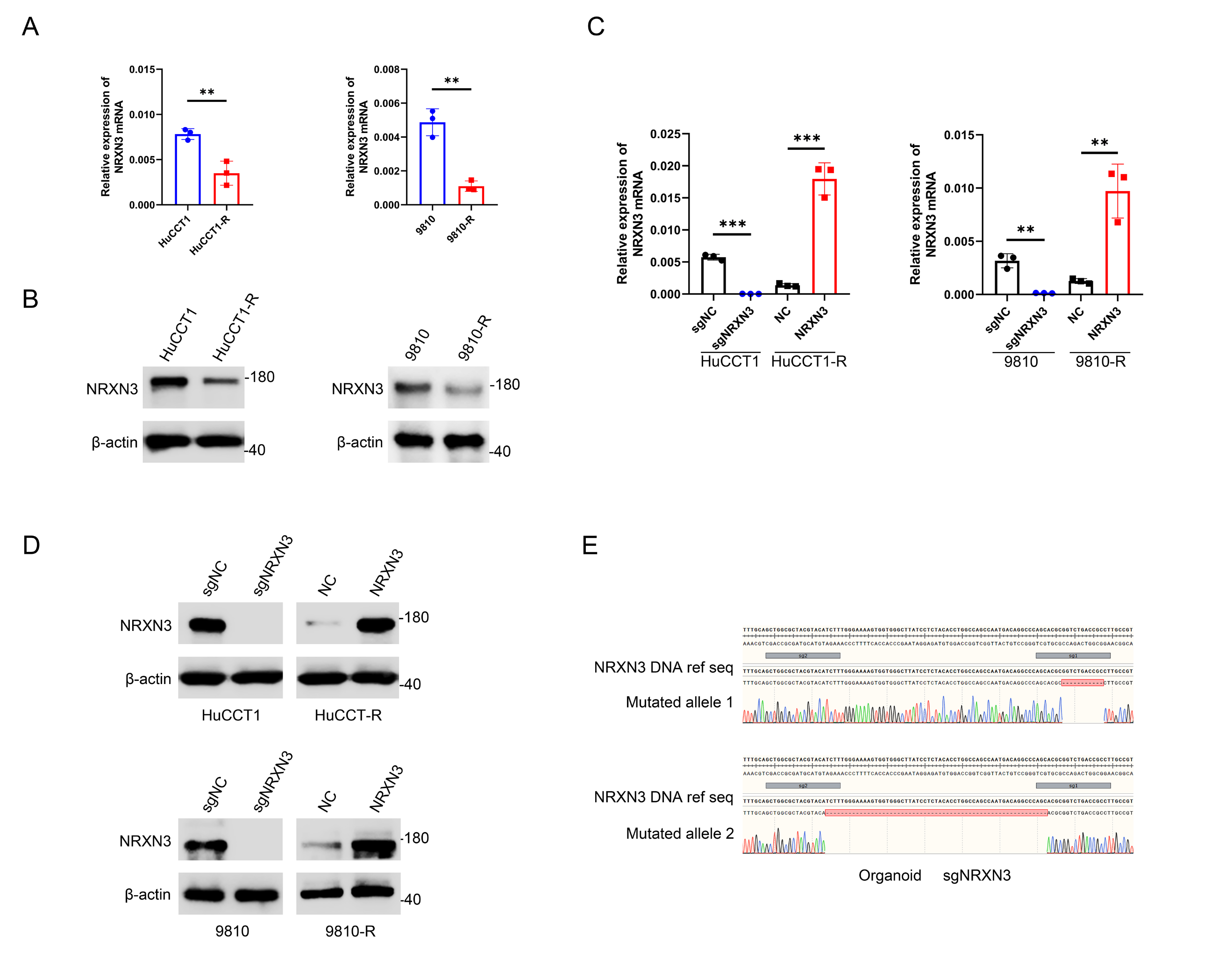
**

**Fig. S2 Genetic modification of NRXN3 in ICC cells and organoids.**

(A) RT-qPCR analysis of NRXN3 expression in HuCCT1, HuCCT1-R, HCCC9810, and HCCC9810-R cells. (B) Immunoblotting analysis of NRXN3 expression in HuCCT1, HuCCT1-R, HCCC9810, and HCCC9810-R cells. (C) RT-qPCR analysis was used to verify the knockout or overexpression efficiency of NRXN3 in ICC cells. (D) Immunoblotting analysis was used to verify the knockout or overexpression efficiency of NRXN3 in ICC cells. (E) Sanger sequencing was used to verify the CRISPR/Cas9-mediated knockout of NRXN3 in the ICC organoid. *P < 0.05, **P < 0.01, and ***P < 0.001. (A, C) Student’s t test.

**Fig. S3**

**
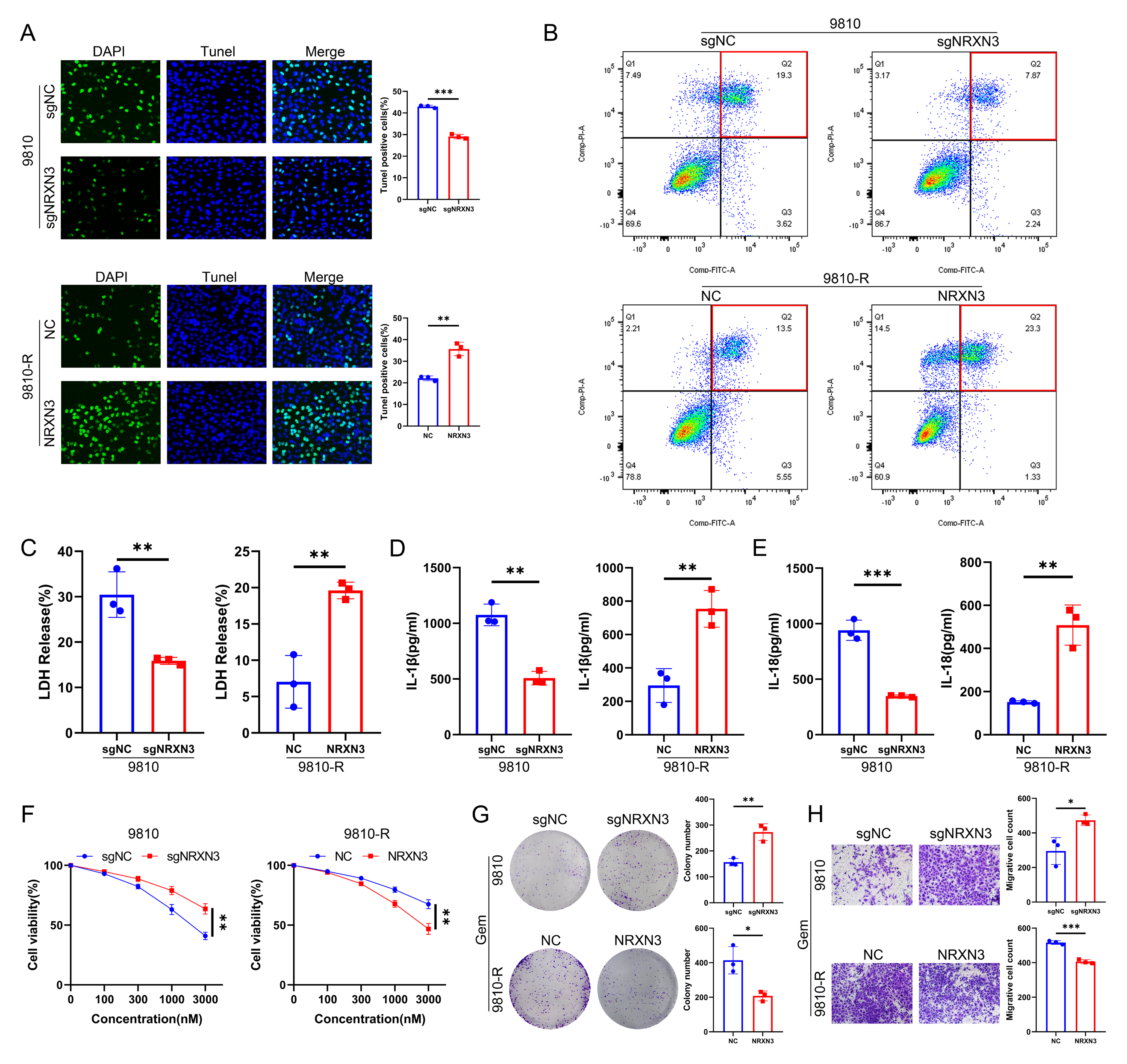
**

**Fig. S3, related to Fig. 2 NRXN3 facilitates gemcitabine-induced pyroptosis and chemosensitivity in ICC.**

(A) Tunel assays of HCCC9810 and HCCC9810-R cells after treatment with gemcitabine. (B) Flow cytometry analysis of HCCC9810 and HCCC9810-R cells treated with gemcitabine and stained with Annexin V-FITC/PI. (C) LDH release assays of HCCC9810 and HCCC9810-R cells after treatment with gemcitabine (20000nM). (D) Measurement of IL-1β release in HCCC9810 and HCCC9810-R cells after treatment with gemcitabine. (E) Measurement of IL-18 release in HCCC9810 and HCCC9810-R cells after treatment with gemcitabine. (F) Cell viability of HCCC9810 and HCCC9810-R cells treated with gemcitabine at different dose for 48h. (G) Colony formation assays of HCCC9810 and HCCC9810-R cells in low concentration of gemcitabine. (H) Migration assays of HCCC9810 and HCCC9810-R cells in the presence of gemcitabine. *P < 0.05, **P < 0.01, and ***P < 0.001. (A, C-H) Student’s t test.

**Fig. S4**

**
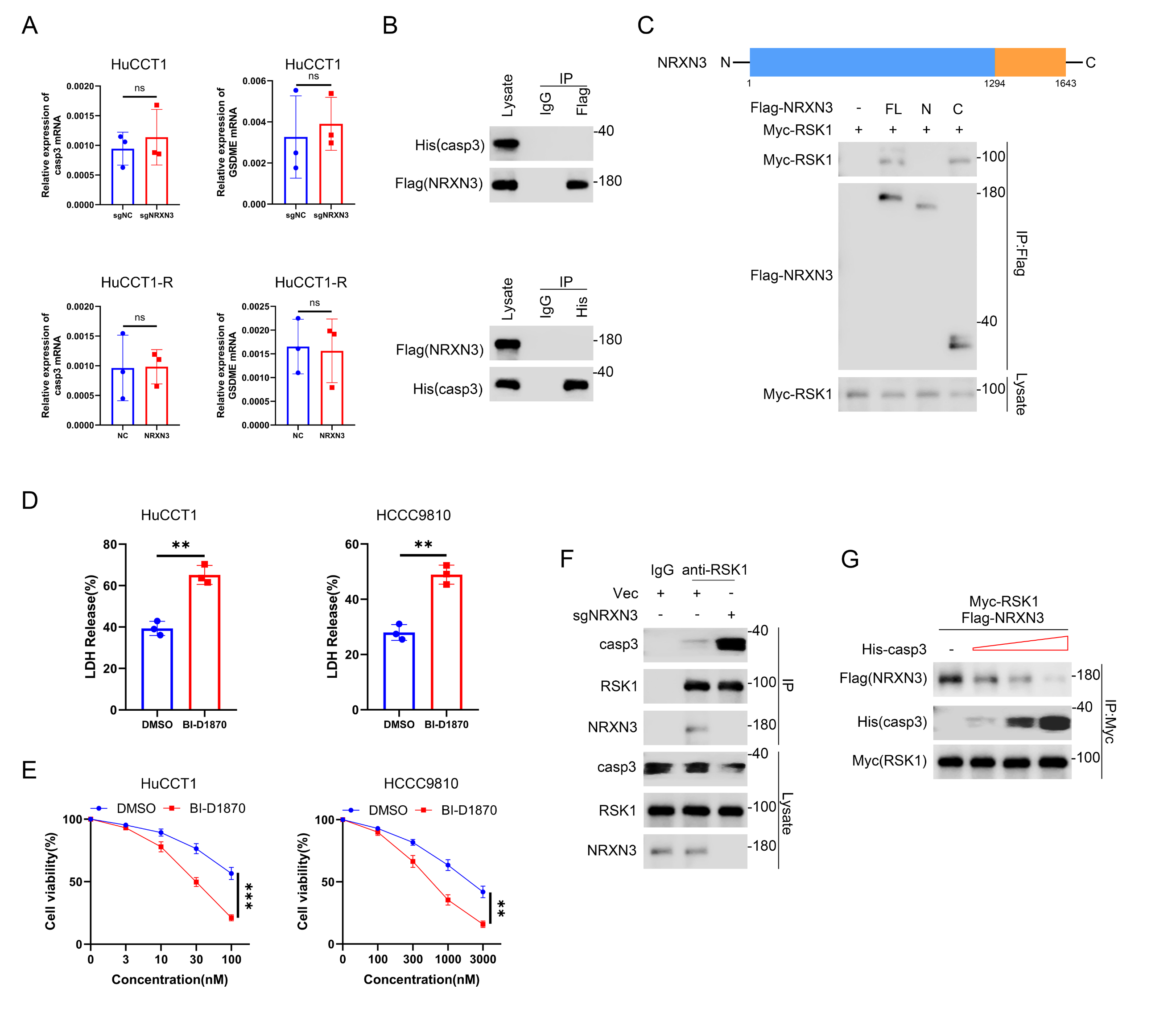
**

**Fig. S4, related to Fig. 3 NRXN3 regulates gemcitabine-induced pyroptosis in ICC cells via blocking caspase-3 interaction with RSK1 and maintaining the stability of caspase-3.**

(A) RT-qPCR analysis of caspase-3 and GSDME mRNA expression in ICC cells with NRXN3 knockout or overexpression. (B) HEK-293T cells were co-transfected with Flag-NRXN3 and His-caspase-3 for 48 hours. Cell lysates were analyzed by IP and IB as indicated. (C) HEK-293T cells were co-transfected with Myc-RSK1 and vector or the indicated Flag-tagged NRXN3 full-length (FL), N-terminus (N) or C-terminus (C) plasmids. Lysates were subjected to anti-Flag immunoprecipitation and IB. (D) LDH release assays of ICC cells treated with BI-D1870 or DMSO in the presence of gemcitabine. (E) Cell viability of BI-D1870- or DMSO-treated ICC cells at different dose of gemcitabine. (F) HuCCT1 cells transduced with vector or sgNRXN3 lentiviruses were subjected to anti-RSK1 immunoprecipitation and IB. (G) HEK-293T cells were singly transfected with Flag-NRXN3, Myc-RSK1 and His-caspase-3. Myc-RSK1- and Flag-NRXN3-expressing lysates were added to all conditions in equal amount. His-caspase-3-expressing lysate was excluded from the first condition and added in incremental amounts to the last three conditions. Lysates were subjected to anti-Myc immunoprecipitation and IB. ^ns^P > 0.05, *P < 0.05, **P < 0.01, and ***P < 0.001. (A, D-E) Student’s t test.

**Fig. S5**

**
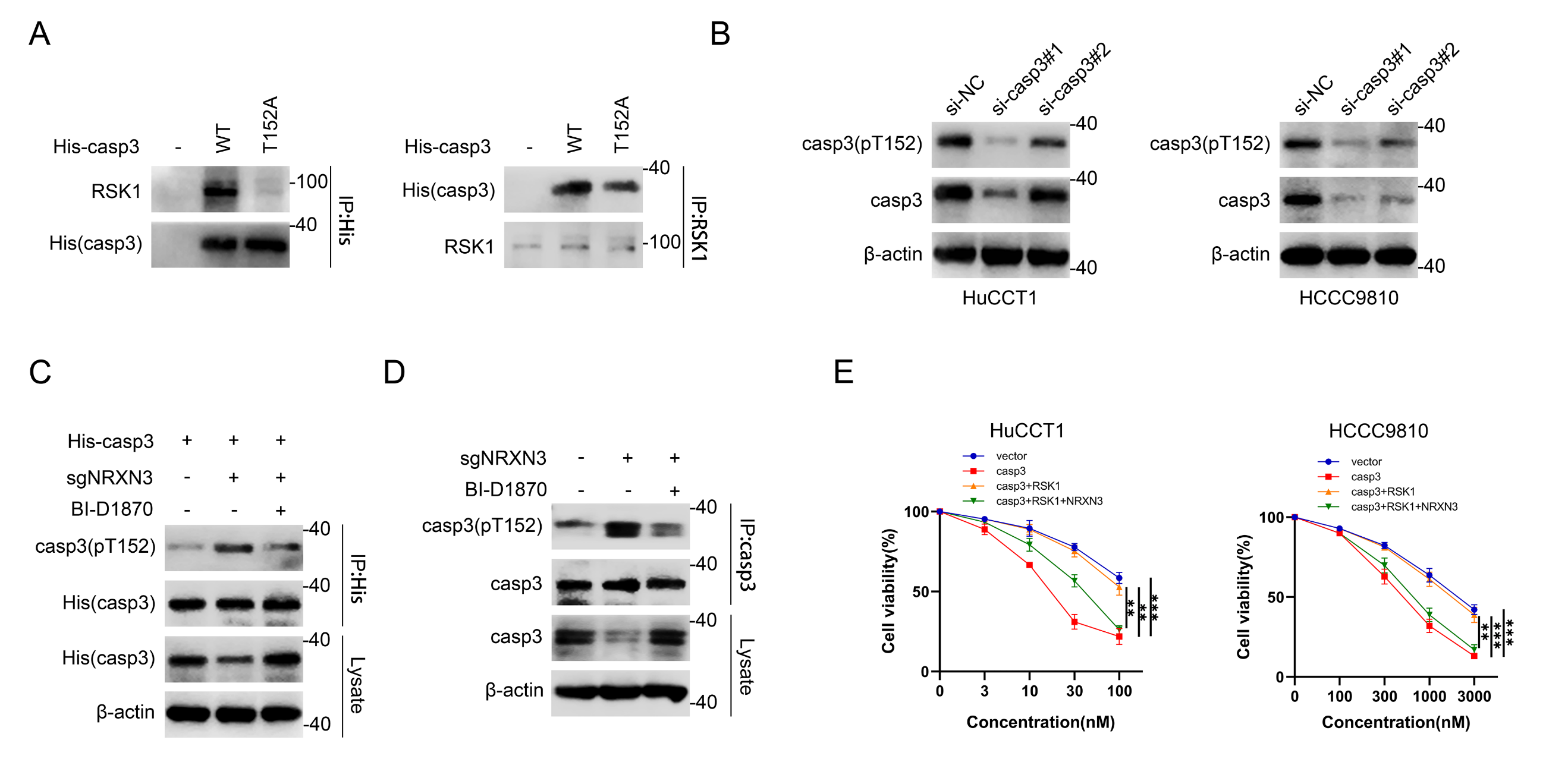
**

**Fig. S5, related to Fig. 4 NRXN3 regulates caspase-3 via RSK1-induced phosphorylation of caspase-3 at T152.**

(A) HEK-293T cells were transfected with vector, His-caspase-3 WT or His-caspase-3 T152A mutant. Lysates were subjected to IP and IB as indicated. (B) HuCCT1 and HCCC9810 cells were transfected with vector or siRNA targeting caspase-3. Lysates were harvested and analyzed by immunoblotting. (C) HuCCT1 cells transduced with vector or sgNRXN3 lentiviruses were transfected with His-caspase-3, followed by BI-D1870 treatment as indicated. Lysates were subjected to anti-His immunoprecipitation and IB analysis. (D) HuCCT1 cells transduced with vector or sgNRXN3 lentiviruses were treated with BI-D1870 as indicated, followed by anti-caspase-3 immunoprecipitation and IB. (E) Cell viability of ICC cells transfected with vector or caspase-3, RSK1 and NRXN3 as indicated at different dose of gemcitabine. *P < 0.05, **P < 0.01, and ***P < 0.001. (E) Student’s t test.

**Fig. S6**

**
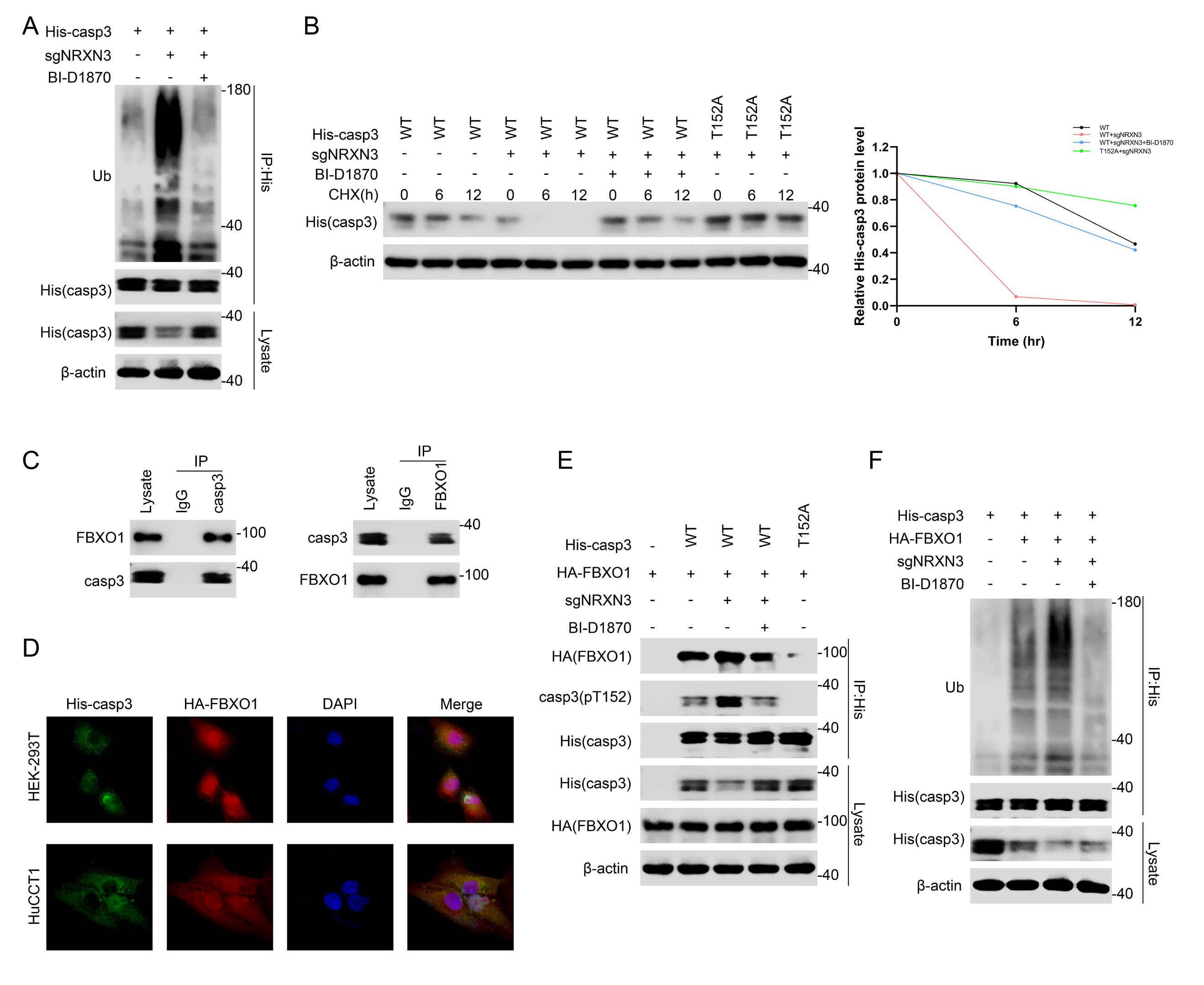
**

**Fig. S6, related to Fig. 5 RSK1-induced caspase-3 phosphorylation at T152 is recognized by FBXO1 for ubiquitination and degradation.**

(A) HuCCT1 cells transduced with vector or sgNRXN3 lentiviruses were transfected with His-caspase-3 and treated with BI-D1870 as indicated. Lysates were subjected to anti-His immunoprecipitation and IB. (B) HuCCT1 cells transduced with vector or sgNRXN3 lentiviruses were transfected with His-caspase-3 WT or T152A mutant and treated with BI-D1870 as indicated, followed by CHX for 0-12 hours. Lysates were used for IB to measure the protein levels of caspase-3. Density of caspase-3 expression was quantified by ImageJ and the relative expression compared with the CHX-untreated condition is plotted. (C) HuCCT1 cell lysates were harvested and analyzed by IP and IB as indicated. (D) Immunofluorescence analysis of His-caspase-3 and HA-FBXO1 in His-caspase-3- and HA-FBXO1-expressing HEK-293T and HuCCT1 cells. (E) HuCCT1 cells transduced with vector or sgNRXN3 lentiviruses were co-transfected with HA-FBXO1 and His-caspase-3 WT or T152A mutant, followed by treatment with DMSO or BI-D1870. Lysates were subjected to anti-His immunoprecipitation and IB. (F) HuCCT1 cells transduced with vector or sgNRXN3 lentiviruses were co-transfected with HA-FBXO1 and His-caspase-3, followed by treatment with DMSO or BI-D1870. Lysates were subjected to anti-His immunoprecipitation and IB.

**Fig. S7**

**
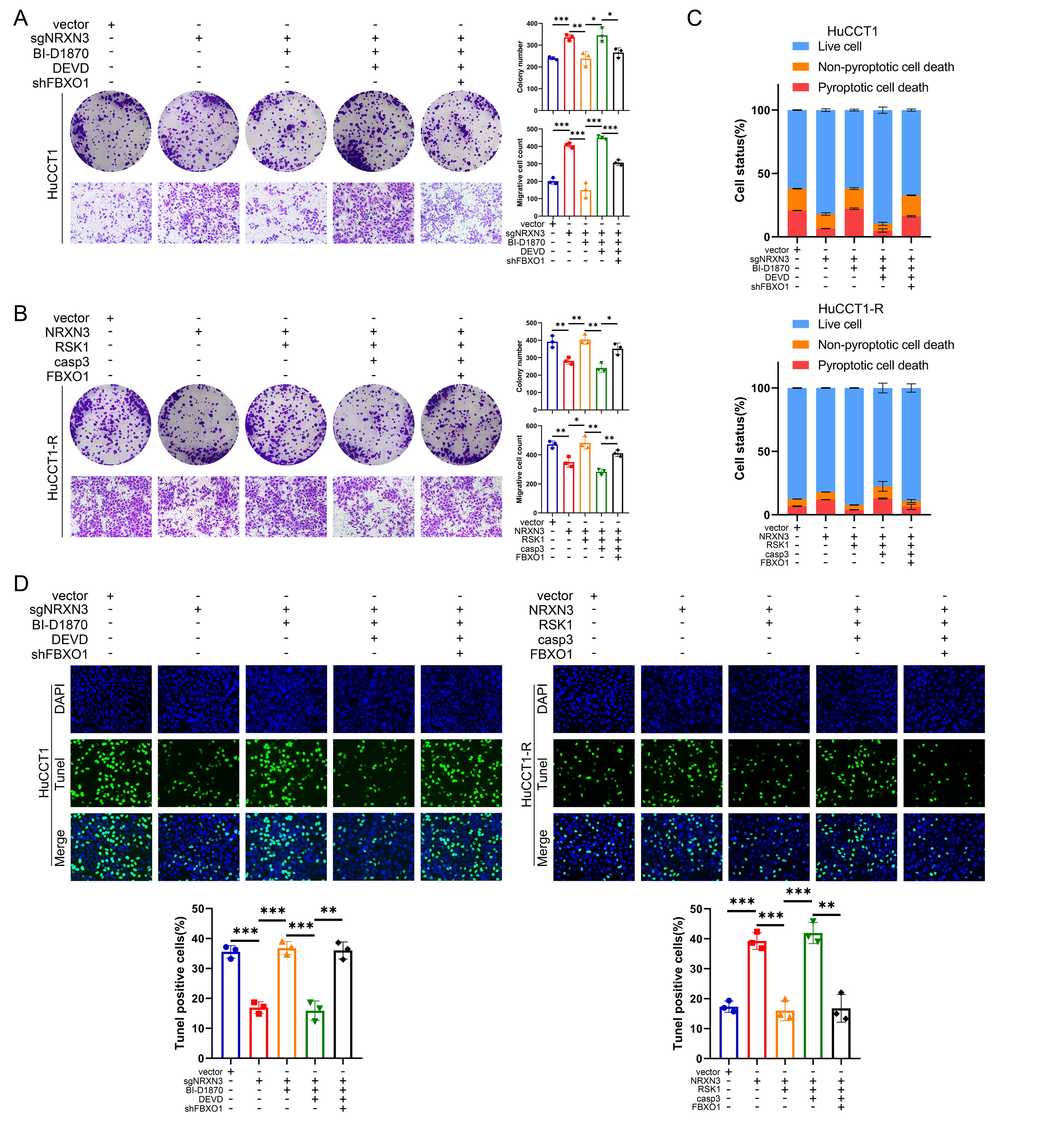
**

**Fig. S7, related to Fig. 6 NRXN3 regulates gemcitabine-induced pyroptosis through RSK1/FBXO1-mediated caspase-3 degradation.**

(A) Colony formation assays and migration assays of HuCCT1 cells in the presence of gemcitabine. HuCCT1 cells transduced with vector or sgNRXN3 and shFBXO1 lentiviruses were treated with BI-D1870 and DEVD as indicated. (B) Colony formation assays and migration assays of HuCCT1-R cells in the presence of gemcitabine. HuCCT1-R cells transduced with vector or NRXN3-overexpressing lentiviruses were co-transfected with RSK1, caspase-3 and FBXO1 as indicated. (C) HuCCT1 cells were treated as in (A) and HuCCT1-R cells were treated as in (B). Flow cytometry analyses were conducted after treatment with gemcitabine. Stacked bar charts show the percentage of cells of each status. Pyroptotic cell death: Annexin V-FITC+/PI+; non-pyroptotic cell death: Annexin V-FITC+/PI- and Annexin V-FITC-/PI+; live cell: Annexin V-FITC-/PI-. (D) HuCCT1 cells were treated as in (A) and HuCCT1-R cells were treated as in (B). Tunel assays were conducted after treatment with gemcitabine. *P < 0.05, **P < 0.01, and ***P < 0.001. (A-B, D) Student’s t test.
