# Supplementary material for: NRXN3 regulates pyroptosis in intrahepatic cholangiocarcinoma via mediating the phospho-dependent ubiquitination and degradation of caspase-3
Source: J Adv Res. 2025 May 3;80:655–69. doi: 10.1016/j.jare.2025.04.040 (PMC12869240; doi:10.1016/j.jare.2025.04.040)
Supplement: Supplementary Data 3 [file mmc3.docx]

**Supplementary Methods**

**CCK8 Assay**

CCK8 assay kit (Dojindo, Japan) was employed to assess cell viability and cytotoxicity. Cells were seeded in 96-well plates at 1×10^3^/well for cell viability detection. In each day of 5 consecutive days, 100μ medium containing 10μ CCK8 reagent was added to each well, and the absorbance at 450 nm was measured with a microplate reader. For cytotoxicity detection or IC_50_ calculation, cells were seeded in 96-well plates at 5×10^3^/well overnight before the indicated treatment.

**Colony Formation Assay**

Cells were seeded in 6-well plates at 1×10^3^/well and incubated at 37°C for 10-14 days accordingly. The medium was refreshed every 4 days. Cell colonies were fixed with 4% paraformaldehyde (Beyotime, China) for 15 min and stained with 0.1% crystal violet (Beyotime, China) for 30 min before they were counted and photographed.

**Transwell Assay**

Transwell assay was used to evaluate the migrative capacity of cells. Briefly, cells of different treatment condition suspended in serum-free medium were seeded in the upper chamber at 2×10^4^/well whereas the lower chamber was filled with medium containing 20% FBS. After 48 or 60 hours, the cells were fixed with 4% paraformaldehyde for 15 min and stained with 0.1% crystal violet for 30 min. The cells on the underside of the membrane were photographed and counted.

**Scanning Electron Microscopy**

Pretreated cells on coverslips were fixed with 2.5% glutaraldehyde for 2 hours. After fixation, cells were post-fixed in 1% osmium tetroxide for 1 hour, and then dehydrated through a graded ethanol series (30% to 100%). Ethanol was replaced with amyl acetate, followed by critical-point drying. The dried samples were mounted on aluminum stubs, sputter-coated with gold-palladium, and examined using Regulus SU8100 (Hitachi, Japan).

**Flow Cytometry Analysis**

Flow cytometry was used to analyze the cell status using Annexin V-FITC/PI Apoptosis Detection Kit (Vazyme). The pretreated cells were harvested and washed with PBS twice. Subsequently, cells were incubated with Annexin V-FITC and PI staining solution for 10 min at room temperature in the shadow. The cells were subjected to FACS Calibur flow cytometer (BD Biosciences, USA) and the results were analyzed with FlowJo v10.5.3.

**TUNEL Assay**

TUNEL Assay Kit (Beyotime) was used to assess cell death. Cells were planted in 24-well plate at 80% confluence and cultured for 24h before fixed in 4% paraformaldehyde for 30 min and permeabilized with 0.3% Triton X-100 for 5 min. The cells were washed twice and incubated with TUNEL solution for 60 min at 37°C in the dark. The nuclei were stained with DAPI (Beyotime).

**Lactate dehydrogenase (LDH) Release Assay**

LDH Release Assay Kit (Beyotime) was purchased to assess cytotoxicity according to the manufacturer’s instruction. Briefly, the culture supernatant of cells exposed to different treatments was collected and the LDH concentration of the supernatant was detected by measuring the absorbance at 490 nm with a microplate reader.

**Detection of IL-1β and IL-18**

The culture supernatant of cells exposed to different treatments was collected and processed with Human IL-1β and IL-18 ELISA Kit (Proteintech) according to the manufacturer’s instruction. The protein standard was used to generated a standard curve and the absorbance at 450 nm was quantified using a microplate reader.

**RNA Extraction and RT-qPCR**

Total RNA was extracted using the FastPure Cell/Tissue Total RNA Isolation Kit (Vazyme, China) according to the manufacturer’s protocol. Reverse transcription was performed using HiScript Q RT SuperMix (Vazyme). RT-qPCR was performed with AceQ qPCR SYBR Green Master Mix (Vazyme). RNA relative expression was calculated using 2^-△△CT^ method with β-Actin as an endogenous control. The sequences of the related primers were listed in Table. S2.

**Total Protein Extraction and Western Blot Analysis**

Total protein was extracted from cell lines or tissues with RIPA Lysis Buffer (Beyotime) or NP-40 Lysis Buffer (Beyotime) supplemented with 1mM PMSF and phosphatase inhibitor. The proteins were separated using SDS-PAGE electrophoresis and transferred to PVDF membranes. The membranes were blocked in Quick Blocking Buffer (NCM Biotech, China) for 30 min and incubated overnight in the indicated primary antibody at 4°C. The membranes were incubated in the corresponding HRP secondary antibody for 2 hours at room temperature.

**Immunohistochemistry and Immunofluorescence**

Tissue microarrays of human samples and sections of mice xenografts were prepared for immunohistochemical staining. The slides were immersed in 3% H2O2 for 5 min at room temperature to block endogenous peroxidase activity and incubated in sodium citrate buffer for 15 min at 95°C for antigen retrieval. After being blocked with 5% normal goat serum for 10 min, the slides were incubated with the indicated antibodies overnight at 4°C, followed by incubation with corresponding secondary antibody for 1 hour at room temperature. Nuclei were stained with DAPI.

For immunofluorescence, cells attached to slides were fixed with 4% paraformaldehyde and permeabilized with Immunostaining Permeabilization Buffer (Beyotime). After washing, the sliders were blocked with 5% BSA in PBS for 1 hour at room temperature and then incubated with the indicated primary antibody overnight at 4°C. The cells were washed three times and incubated with corresponding secondary antibody for 1 hour at room temperature in the dark. The cells were washed twice and the nuclei were stained with DAPI.
